# USP4 promotes the proliferation, migration, and invasion of esophageal squamous cell carcinoma by targeting TAK1

**DOI:** 10.1038/s41419-023-06259-0

**Published:** 2023-11-10

**Authors:** Hongdian Zhang, Youming Han, Wanyi Xiao, Yongyin Gao, Zhilin Sui, Peng Ren, Fanbiao Meng, Peng Tang, Zhentao Yu

**Affiliations:** 1 Tianjin Medical University Cancer Institute and Hospital, National Clinical Research Center for Cancer, Tianjin’s Clinical Research Center for Cancer, Key Laboratory of Digestive Cancer of Tianjin, Tianjin, 300060 China; 2 Binhai Hospital of Tianjin Medical University General Hospital, Tianjin, 300456 China; 3 National Cancer Center, National Clinical Research Center for Cancer, Cancer Hospital & Shenzhen Hospital, Chinese Academy of Medical Sciences and PeKing Union Medical College, Shenzhen, 518116 China

**Keywords:** Metastasis, Oesophageal cancer, Oncogenesis

## Abstract

Ubiquitin-specific protease 4 (USP4) represents a potential oncogene involved in various human cancers. Nevertheless, the biological roles and precise mechanism of USP4 in esophageal squamous cell carcinoma (ESCC) progression are not understood. Here, USP4 expression was found to be markedly upregulated in ESCC tumor tissues and cells. Loss- and gain-of-function assays suggested that USP4 silencing inhibited ESCC cell proliferation, migration, and invasion, while USP4 overexpression promoted these behaviors. Consistently, USP4 silencing repressed tumor growth and metastasis in an ESCC nude mouse model in vivo. As a target molecule of USP4, transforming growth factor-β-activated kinase 1 (TAK1) also showed high expression in ESCC. Moreover, we observed that USP4 specifically interacted with TAK1 and stabilized TAK1 protein levels via deubiquitination in ESCC cells. Importantly, USP4 promotes ESCC proliferation, migration, and invasion via the MEK/ERK signaling pathway and can be inhibited by U0126. Neutral red (NR), an inhibitor of USP4 can suppress ESCC progression in vitro and in vivo. Overall, this study revealed that USP4/TAK1 plays crucial roles in ESCC progression by modulating proliferation, migration, and invasion, and USP4 might be a potential therapeutic target in ESCC.

## Introduction

Esophageal squamous cell carcinoma (ESCC) is one of the most common lethal esophageal malignancies that arises from abnormal hyperplasia of the esophageal squamous epithelium [1]. Because of the lack of overt early clinical symptoms in ESCC, most cases are commonly diagnosed at an advanced stage [2]. Although multiple strategies such as surgery, radiation, chemotherapy, and immunotherapy have been used for the treatment of ESCC, patient prognosis remains unfavorable because of the high rates of metastasis, recurrence, and drug resistance [3, 4]. Reportedly, molecular targeted therapy has been applied as a promising therapeutic method with less toxicity [5]. Nonetheless, the outcomes in terms of long-term survival are not satisfactory [6]. Therefore, clarification of the mechanism of ESCC and identification of novel target molecules are essential for effective ESCC treatment.

Ubiquitination is one of the major pathways of protein degradation and is coordinated by the action of ubiquitylating and deubiquitylating enzymes (DUBs) [7]. Accumulating evidence has established that ubiquitination and deubiquitination systems are closely related to cancer progression and may contribute to potential therapeutic targets for the treatment of cancers [8, 9]. Ubiquitin-specific protease 4 (USP4), which belongs to the USP family, was the first DUB identified in mammalian cells [10]. It was previously demonstrated that USP4 has an important function in DNA repair due to its autodeubiquitination ability [11]. It also participates in numerous signaling pathways, modulates various human pathological and physiological processes, and acts as a potential oncogene in diverse human cancer types by inhibiting p53 function [12] or promoting epithelial–mesenchymal transition (EMT) [13]. Notably, USP4 was also identified in esophageal cancer (EC) and is positively associated with the pathological grade and prognosis [14]. Nevertheless, the biological function and underlying molecular mechanism of USP4 in ESCC remain unclear.

In this study, the upregulation of USP4 was further validated in ESCC patients, and its correlation with prognosis was identified. Moreover, the function and mechanism of USP4 in the progression of ESCC were investigated in vitro and in vivo after USP4 silencing or overexpression. Taken together, the current results indicated that USP4 has pro-oncogenic effects in ESCC and may serve as a potential therapeutic target for ESCC treatment.

## Materials and methods

### Patients, human tissues, and cell lines

A total of 178 ESCC patients (146 males and 32 females) who underwent surgical treatment with complete follow-up data in our hospital from January 2009 to December 2010 were enrolled. No patients were administered any preoperative radiotherapy, chemotherapy, targeted therapy, immunotherapy, or other related antitumor therapies. The utilized tissue microarray (TMA) chips were prepared at Shanghai Outdo Biotech Co., Ltd. (Shanghai, China). The patients’ detailed information is summarized in Table 1. In total, 30 paired fresh ESCC and adjacent noncancerous tissue specimens were collected for quantitative real-time PCR (qRT-PCR) and Western blotting analysis. Informed consent was obtained from each patient, and this study was approved by the Research Ethics Committee of the Cancer Hospital of Tianjin Medical University (Batch number: bc2021340).

**Table 1:** Relationship between USP4 expression and tumor characteristics in patients with ESCC.

Clinicopathologic features	Cases	USP4 expression		*x*^*2*^	*P* value
		Low	High		
*Gender*				1.165	0.281
Male	146	46 (31.5%)	100 (68.5%)		
Female	32	7 (21.9%)	25 (78.1%)		
*Age (years)*				1.166	0.280
≤68	83	28 (33.7%)	55 (66.3%)		
>68	95	25 (26.3%)	70 (73.7%)		
*Smoking history*				2.003	0.157
Yes	121	32 (26.4%)	89 (73.6%)		
None	57	21 (36.8%)	36 (63.2%)		
*Tumor location*				0.641	0.726
Upper	7	2 (28.6%)	5 (71.4%)		
Middle	149	46 (30.9%)	103 (69.1%)		
Lower	22	5 (22.7%)	17 (77.3%)		
*Tumor size (cm)*				5.368	**0.021**
<3.5	74	29 (39.2%)	45 (60.8%)		
≥3.5	104	24 (23.1%)	80 (76.9%)		
*Histological type*				1.297	0.523
G1	9	3 (33.3%)	6 (66.7%)		
G2	133	42 (31.6%)	91 (68.4%)		
G3	36	8 (22.2%)	28 (77.8%)		
*Invasion depth*				7.118	**0.028**
pT1–2	30	15 (50.0%)	15 (50.0%)		
pT3	96	24 (25.0%)	72 (75.0%)		
pT4	52	14 (26.9%)	38 (73.1%)		
*Lymph node metastasis*				5.713	**0.017**
No	107	39 (36.4%)	68 (63.6%)		
Yes	71	14 (19.7%)	57 (80.3%)		
*TNM staging*				2.571	0.109
I–II	68	25 (36.8%)	43 (63.2%)		
III	110	28 (25.5%)	82 (74.5%)		

Bold is used to emphasize that the value is statistically significant.

*ESCC* esophageal squamous cell carcinoma, *USP4* ubiquitin-specific protease 4, *G1* well differentiated, *G2* moderately differentiated, *G3* poorly differentiated/undifferentiated, *TNM* tumor-nodes metastasis.

Human EC (KYSE150, EC109, KYSE140, KYSE450, KYSE180, and TE-1) and human immortalized esophageal epithelial (Het-1A) cells were provided by the Cell Bank of the Chinese Academy of Sciences (Shanghai, China). All cells were cultured in RPMI 1640 (Gibco, USA) containing 10% FBS (BI, USA) and maintained at 37 °C in a 5% CO_2_ humidified incubator. Cell line authentication was performed using short tandem repeat (STR) DNA profiling.

### Immunohistochemical (IHC) staining

The tissue samples were embedded in paraffin and sliced into 5-μm-thick sections. After deparaffinization at 70 °C, clearing with xylene and hydration with gradient alcohol were carried out. After antigen repair in the EDTA buffer, endogenous peroxidases were inactivated with 3% H_2_O_2_. Subsequently, the sections were blocked with 5% goat serum and incubated with primary antibodies targeting USP4 (1:100, Proteintech) and TAK1 (1:100, Proteintech) at 4 °C overnight. On the following day, the sections were incubated with biotin-linked secondary antibodies. DAB solution was used for color development, and the sections were counterstained with hematoxylin and dehydrated in xylene solution. Finally, samples were analyzed by microscopy and scored according to the proportion of positive cells and staining intensity. The staining intensity was scored as follows: 0 (no staining), 1 (light yellow), 2 (light brown), or 3 (brown), and the proportion of positive cells was scored as follows: 0, <10%; 1, 10–25%; 2, 26–50%; 3, 51–75%; and 4, >75%. The pathological scores were calculated according to a previous study [15].

### Lentiviral, small interfering RNA, and plasmid transfection

For stable cell line construction, lentiviral particles with USP4 knockdown or USP4 overexpression plasmids were purchased from GeneChem (Shanghai, China). KYSE150 and KYSE180 cells were infected with a lentiviral solution to produce stable USP4 knockdown, USP4 overexpression, and matched control cell lines.

For transient transfection, small interfering RNA (siRNA) specifically targeting TAK1 was synthesized by GenePharm (Shanghai, China). The TAK1 overexpression plasmid and HA-Ub, HA-Ub-K48R, and HA-Ub-K63R plasmids constructed with pcDNA3.1 were purchased from GeneChem (Shanghai, China). Cells were transfected with siRNA or the indicated plasmids using Lipofectamine 2000 (Invitrogen, Life Technologies) according to the manufacturer’s instructions. The sequences of USP4 shRNAs and TAK1 siRNA are shown in Supplementary Table 1.

### Quantitative real-time PCR (qRT‒PCR)

When the KYSE150 and KYSE180 cells reached 80% confluence, they were harvested, and the total RNA was extracted with TRIzol reagent (Invitrogen, USA). Subsequently, the extracted RNA was reverse-transcribed to synthesize the corresponding cDNA using the PrimeScript RT Reagent Kit (TaKaRa Bio, Japan) according to the manufacturer’s instructions. PCR amplification was performed using an ABI 7500 Sequence Detection System with SYBR Premix Ex Taq II Reagent (TaKaRa Bio, Japan). The 2^−^^△△CT^ method was carried out for data analysis, with GAPDH used as a control gene. The primers used in the detection are listed in Supplementary Table 2.

### Western blotting

After cell lysis, total protein was obtained from ESCC tumor tissues or ESCC cells. Subsequently, the BCA protein assay kit (Pierce, USA) was utilized for protein quantitation. Equal amounts of protein were separated by 10% SDS-PAGE, transferred onto PVDF membranes, and blocked with TBST containing 5% skimmed milk for 1 h at ambient temperature. Subsequently, the membrane was probed with primary (4 °C overnight) and then secondary (room temperature for 2 h) antibodies (Supplementary Table 3), followed by development with enhanced chemiluminescence reagents (Millipore) to visualize the immunoreactive bands. Protein signals were captured with a chemiluminescence imager. ImageJ was used to measure and analyze the optical density. The original western blots were shown in Supplementary Material.

### Colony formation assay, cell counting kit-8 (CCK-8) assay, and EdU incorporation assay

Colony formation and CCK-8 assays were conducted according to our published protocol [16]. The EdU assay was performed using a Click-iT EdU Kit (BeyoClick™ EdU-555). In brief, KYSE150 and KYSE180 cells were incubated with 2 × EdU for 2 h, and then the cells were fixed with 1 ml paraformaldehyde, permeabilized with Triton X-100 for 15 min, and incubated with 500 μl Click reaction cocktail for 30 min. The cells were stained with Hoechst 33342 and mounted for microscopic imaging.

### Wound healing assay

The wound-healing assay was performed as previously described [17]. ESCC cells were added to a 6-well plate and cultured under routine conditions. At 70–80% confluency, cells were further grown in a low-serum medium and scratched using a 10 μl pipette tip. After removing floating cells, the wounded areas were imaged by fluorescence microscopy at 0, and 24 h time points to determine the cell migration rate of each group.

### Transwell assay

Cell invasion was assessed using Transwell chambers with 8 μm pores precoated with Matrigel (BD Biosciences) for 4 h. Prior to the assay, ESCC cells were pretreated for 24 h in FBS-free RPMI 1640 medium. Then 200 μL of FBS-free cell suspension (2‒5 × 10^5^ cells/ml) was placed in the top Matrigel-coated Transwell chamber, and 500 μL 20% FBS RPMI 1640 medium was placed in the bottom compartment and maintained at 37 °C. After incubation for 24 h, invasive cells on the lower surface of the membrane were fixed with 4% paraformaldehyde and stained with crystal violet. The number of invading cells was quantified using a fluorescence microscope (Olympus, Japan). Cell migration assessment was performed following the above steps, except that the top chambers were not coated with Matrigel.

### In vivo tumor growth and metastasis assays

All animal experiments were approved by the Institutional Animal Care and Use Committee (IACUC) of Tianjin Medical University Cancer Institute and Hospital (Approval No. PMLF-2021113). Five- to 6-week-old male BALB/c nude mice and severe combined immune deficiency/beige (SCID/Beige) mice were provided by Beijing HFK Bioscience (Beijing, China). Exponentially growing experimental or control cells transfected with lentivirus were randomly administered at 5 × 10^6^/mouse by subcutaneous injection into the right lateral axillary region of BALB/c nude mice (*n* = 5 per group). To evaluate the inhibitory effect of NR (Sigma-Aldrich) or U0126 (Sigma-Aldrich) on in vivo tumor growth, each group was intraperitoneally injected with either PBS (as a control), NR (50 mg/kg), or U0126 (15 mg/kg) every 3 days. After tumor formation, mouse body weights and tumor sizes were recorded at 5-day intervals. Tumor volume was calculated according to the formula: (length × width^2^)/2. Euthanasia was carried out four weeks post-cell injection, and xenograft tumors were extracted, weighed, photographed, and fixed with paraformaldehyde for immunohistochemical staining.

Metastasis experiments were performed as previously described [16]. ESCC cells (5 × 10^5^) labeled with luciferase were injected into male SCID/Beige mice via the tail vein. After 2 months, in vivo, fluorescence imaging was performed by using a bioluminescence imaging system. Euthanasia was carried out, and lung samples were obtained and fixed with paraformaldehyde containing picric acid, followed by H&E staining to monitor metastatic nodules.

### Protein half-life assays

The protein synthesis inhibitor cycloheximide (CHX, 100 µg/ml, Sigma-Aldrich) was used to treat KYSE150 and KYSE180 cells and evaluate protein stability. Total proteins were extracted from cells at 0, 3, 6, and 9 h post-CHX administration. Western blotting was carried out to evaluate USP4 and TAK1 protein levels.

### Mass spectrometry (MS)

Stably transfected KYSE180 cells were incubated with 10 mM protease inhibitor MG132 (HY-13259, MCE) for another 4 h. The cell lysates were immunoprecipitated with anti-Flag M2 affinity gel (A2220, Sigma-Aldrich). This process was followed by SDS-PAGE separation and Coomassie blue staining. The entire lane was excised and digested with trypsin. Then, the immunoprecipitates were analyzed with liquid chromatography‒tandem mass spectrometry (LC‒MS/MS). The identification of the peptide mixtures was performed by Ollwegene Technology (Beijing, China).

### Co-immunoprecipitation (Co-IP) and ubiquitylation assay

For Co-IP experiments, total cell lysates were extracted using NP-40 lysis buffer containing MG-132, and the supernatant was collected. For immunoprecipitation, cell lysates were incubated with the indicated antibody at 4 °C overnight, followed by treatment with protein A/G agarose for 6 h. Then, the immunoprecipitates were washed three times and analyzed by Western blotting.

For ubiquitination assays, cells were transfected with the indicated plasmids for 48 h, and then cells were lysed with ubiquitination buffer containing 1% SDS and boiled at 95 °C. The denatured cell lysates were diluted with SDS-negative RIPA buffer to reduce SDS to 0.2% and were then subjected to Co-IP followed by Western blotting with anti-HA or anti-Ub antibody, and the antibodies used in the experiment are shown in Supplementary Table 3.

### Statistical analysis

SPSS 21.0 and GraphPad 9.0 were utilized for data analysis. The data were presented as the mean ± standard deviation (SD) and were compared by Student’s *t test*, one-way analysis of variance (ANOVA), or the Mann‒Whitney *U* test, as appropriate. The correlations between USP4 expression and clinicopathological factors were assessed by the Pearson *χ*2 test or Fisher’s exact test. Survival analysis was performed using the Kaplan‒Meier method and compared via the log-rank test. *P* < 0.05 was considered statistically significant.

## Results

### USP4 is highly expressed in ESCC tissues and cells

**Fig. 1:**
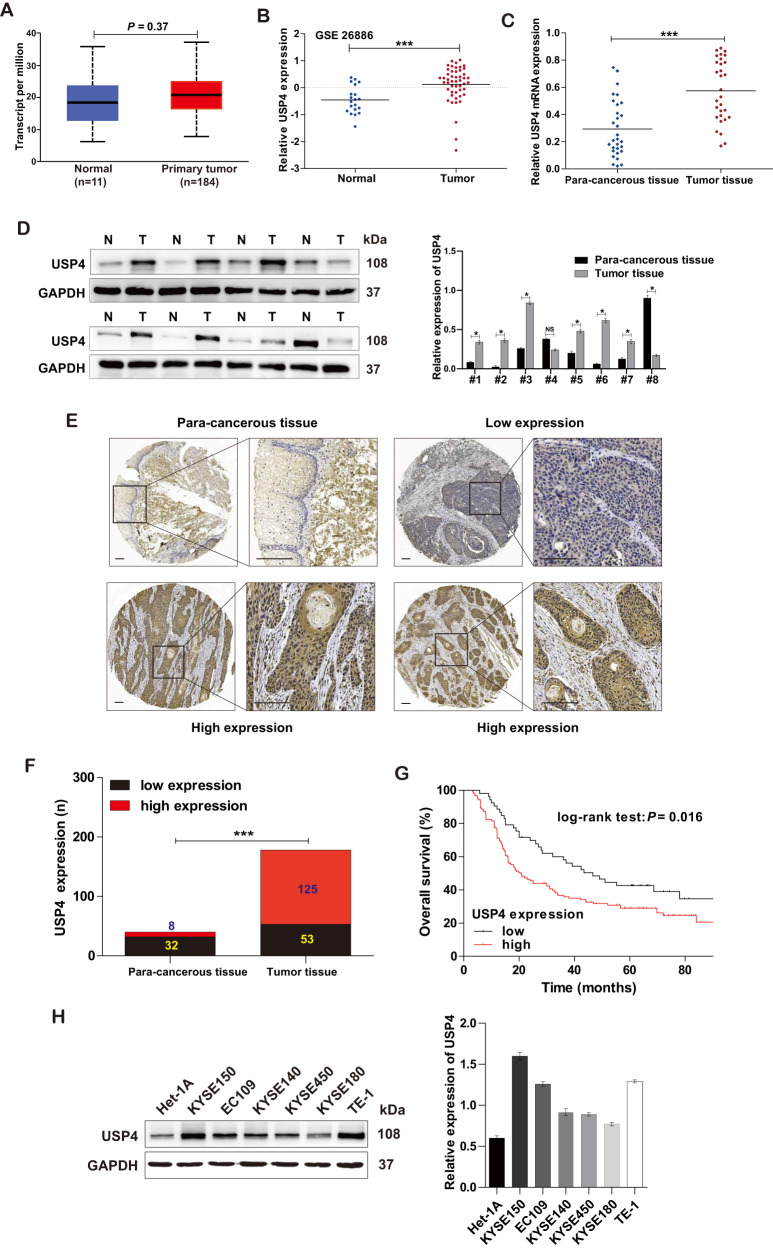
USP4 was highly expressed in ESCC. **A**, **B** Assessment and quantification of USP4 expression in EC primary tumor and normal tissues from the TCGA database and GSE26886 dataset. **C** USP4 mRNA levels in tumor and para-cancerous tissue specimens were measured by qRT-PCR (*n* = 30). **D** The expression of USP4 protein in ESCC patients was assessed by Western blotting (*n* = 30). **E**, **F** Representative images and quantitation of USP4 expression in ESCC (*n* = 178) and para-cancerous (*n* = 40) tissues. **G** Kaplan‒Meier analysis of the overall survival rate in ESCC patients based on USP4 expression. **H** Western blotting analysis of USP4 protein expression in ESCC (KYSE150, EC109, KYSE140, KYSE450, KYSE180, and TE-1) and human immortalized esophageal epithelial (Het-1A) cells. *P < 0.05, ***P < 0.001.

To clarify the role of USP4 in ESCC, the expression of USP4 in EC patients was analyzed using the TCGA database (https://tcga-data.nci.nih.gov/tcga/) and the GSE26886 dataset (https://www.ncbi.nlm.nih.gov/gds/?term=GSE26886). USP4 expression was slightly upregulated in primary tumor tissues in comparison with normal tissues (Fig. 1A, B). Next, we validated the significant upregulation of USP4 in fresh ESCC tissues versus adjacent para-cancerous tissues by qRT-PCR and Western blotting (Fig. 1C, D). Furthermore, IHC staining of TMA sections including ESCC tissue and matched para-cancerous tissue samples demonstrated that USP4 was expressed at higher levels in ESCC tissues than in the corresponding para-cancerous tissues (Fig. 1E). Moreover, high expression of USP4 was predominantly detected in 70.2% (125/178) of the ESCC tumor specimens, whereas only 20.0% (8/40) of the corresponding para-cancerous tissues showed high expression (*P* < 0.001) (Fig. 1F). Next, the relationship between USP4 expression and clinicopathological features in ESCC patients was analyzed. Statistical analysis revealed that high expression of USP4 was correlated with tumor size, invasion depth, and lymph node metastasis (Table 1). Additionally, Kaplan‒Meier survival analysis suggested that USP4 expression had a negative correlation with overall survival in ESCC patients (*P* = 0.016, Fig. 1G).

In addition, the protein levels of USP4 in ESCC (KYSE150, EC109, KYSE140, KYSE450, KYSE180, and TE-1) cell lines were significantly increased compared with those in human immortalized esophageal epithelial (Het-1A) cells (Fig. 1H). Altogether, these results suggest a critical role of USP4 in the progression of ESCC.

### USP4 induces ESCC cell proliferation and tumor growth

**Fig. 2:**
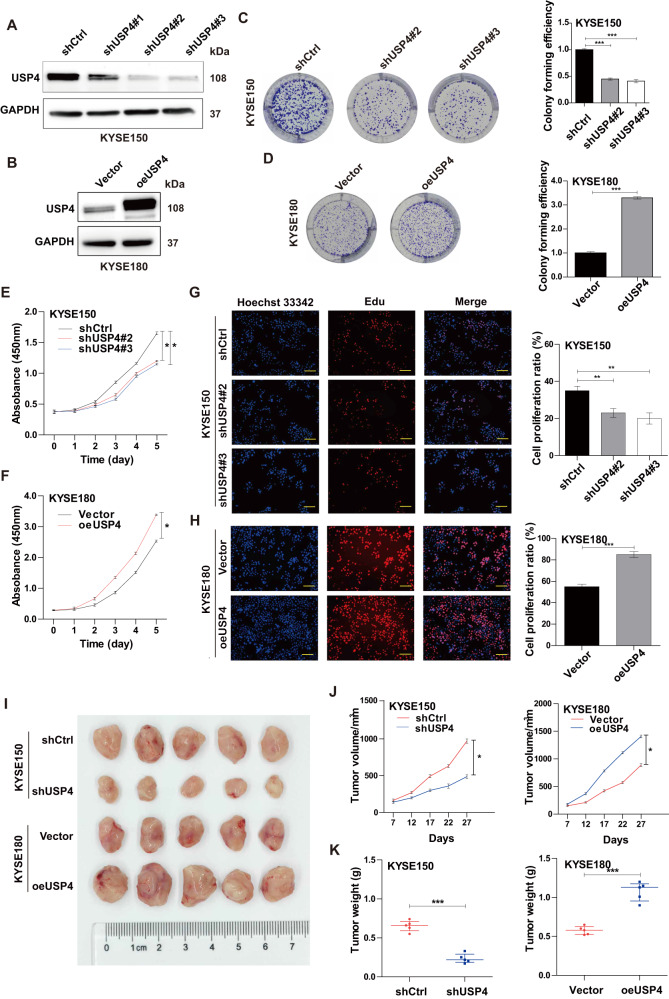
USP4 promotes ESCC cell proliferation in vitro and in vivo. **A**, **B** USP4 protein levels in KYSE150 and KYSE180 cells after transfection with lentiviral solution were determined by Western blotting. **C**, **D** Colony formation was detected by single-cell clone assay. Representative images are shown (left panel), and the results were analyzed statistically (right panel). **E**–**H** Cell proliferation of transfected KYSE150 and KYSE180 cell lines was measured by CCK-8 assays (**E**, **F**) and EdU incorporation (**G**, **H**). **I** Representative images of nude mice and tumors at day 27 after inoculation of KYSE150 cells transfected with shCtrl or shUSP4 lentiviruses and KYSE180 cells transfected with vector or oeUSP4 lentiviruses (*n* = 5). **J** Quantitative analysis of tumor volume with the time lapse from day 7 onwards. **K** Comparison of tumor weight in the control and experimental groups. *P < 0.05, **P < 0.01, ***P < 0.001.

To further confirm the biological function of USP4 in ESCC progression, USP4 knockdown and overexpression cell models were constructed in KYSE150 and KYSE180 cells, respectively. The mRNA and protein levels of USP4 in KYSE150 cells were reduced after transfection with shUSP4#1, shUSP4#2, and shUSP4#3, especially shUSP4#2 and shUSP4#3 (all *P* < 0.001) (Fig. 2A and Supplementary Fig. 1A, B). In contrast, USP4 overexpression increased USP4 mRNA and protein levels in KYSE180 cells (all *P* < 0.001) (Fig. 2B and Supplementary Fig. 1C, D). The above data indicated successful USP4 knockdown or overexpression in cell models. Colony formation assays demonstrated that shUSP4#2 and shUSP4#3 significantly inhibited the proliferation of KYSE150 cells (Fig. 2C) and that USP4 overexpression significantly promoted the proliferation of KYSE180 cells (Fig. 2D). Similar results were observed using the CCK-8 assay (Fig. 2E, F). Additionally, EdU incorporation further confirmed the suppressive effects of USP4 knockdown and the promoting effects of USP4 overexpression on proliferation ability (Fig. 2G, H).

Next, tumor xenograft models were established in nude mice to determine the effects of USP4 on ESCC growth. As shown in Fig. 2I, the isolated tumors in the shUSP4 group were smaller than those in the shCtrl group, and the tumors in the oeUSP4 group were significantly larger than those in the vector group. Furthermore, the tumor volume and weight were significantly decreased in the shUSP4 group and increased in the oeUSP4 group versus the corresponding control groups (Fig. 2J, K). The results of IHC staining demonstrated that USP4 expression in xenograft tumors was decreased by USP4 downregulation but increased by USP4 overexpression (Supplementary Fig. 1E). Together, these findings indicate that USP4 promotes cell proliferation and tumor growth in ESCC.

### USP4 promotes ESCC cell migration and invasion

**Fig. 3:**
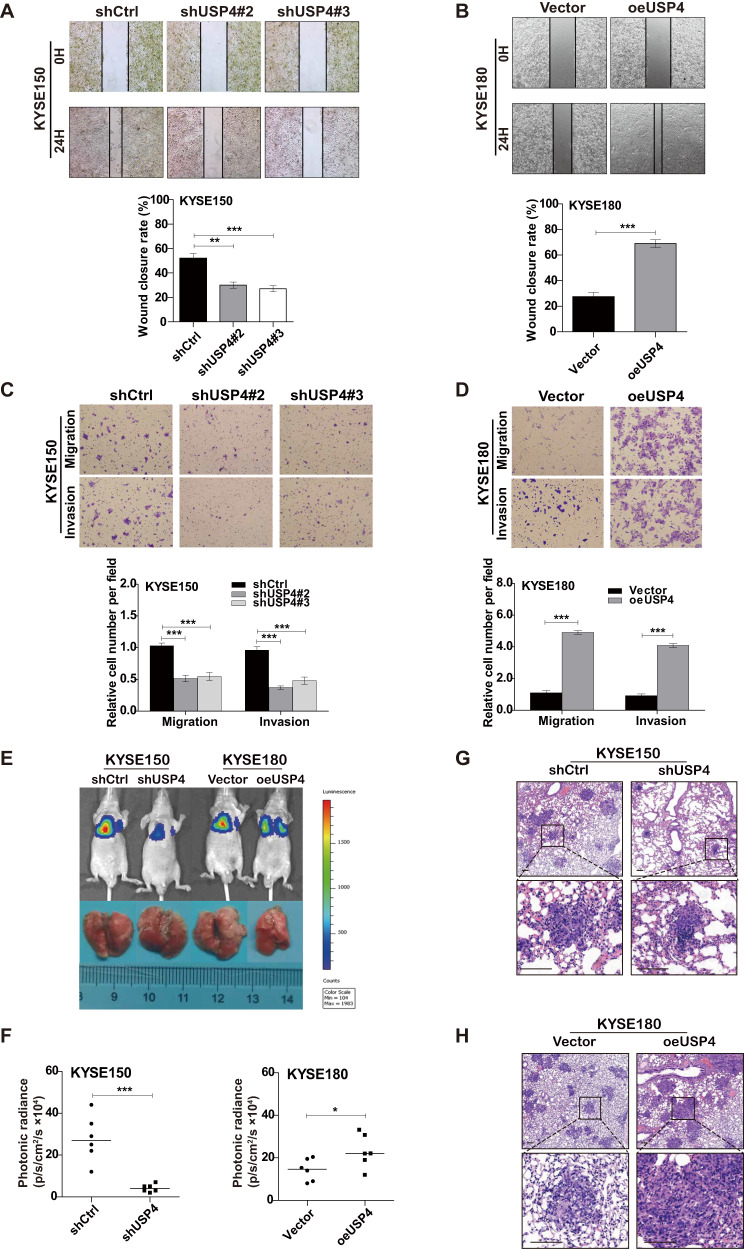
USP4 promotes ESCC cell migration and invasion in vitro and metastasis in vivo. **A**–**D** The migration and invasion of KYSE150 cells with USP4 knockdown and KYSE180 cells with USP4 overexpression were evaluated using wound healing (**A**, **B**) and Transwell (**C**, **D**) assays (*n* = 3). **E**, **F** ESCC cells with stable USP4 knockdown or overexpression and the corresponding control cells were injected into the tail vein of SCID/Beige mice, the lungs were harvested, and the number of nodules was counted. The luminescence signal with the signal intensity is indicated by the scale. **G**, **H** Tumor nodules in lung tissues were stained with H&E. *P < 0.05, **P < 0.01, ***P < 0.001.

In the wound healing assay, the migration capability of KYSE150 cells was attenuated in cells transfected with shUSP4 compared to cells transfected with shCtrl (Fig. 3A), whereas USP4 overexpression induced the opposite effects in KYSE180 cells (Fig. 3B). Similar results were obtained from Transwell assays, which also suggested that cell invasion was markedly attenuated by USP4 silencing in KYSE150 cells but was increased by USP4 overexpression in KYSE180 cells (Fig. 3C, D).

In vivo, 5 × 10^5^ control and USP4-knockdown KYSE150 cells or USP4-overexpressing KYSE180 cells were administered intravenously to mice. As shown in Fig. 3E, the metastatic lung nodules were visualized through an in vivo luciferase imaging system. Consistent with the above findings, the number of lung nodules was significantly decreased by USP4 silencing, while the number of lung nodules was strikingly increased by USP4 overexpression (Fig. 3E, F). Hematoxylin-eosin (H&E) staining was carried out to assess lung nodules in mice based on the tissue structure and nuclei, and the staining results also showed that USP4 knockdown inhibited the lung metastasis of KYSE150 cells, while USP4 overexpression in KYSE180 cells promoted lung metastasis in vivo (Fig. 3G, H).

Collectively, these data suggest that USP4 significantly promotes ESCC cell migration and invasion in vitro and metastasis in vivo.

### TAK1 is positively regulated by USP4

**Fig. 4:**
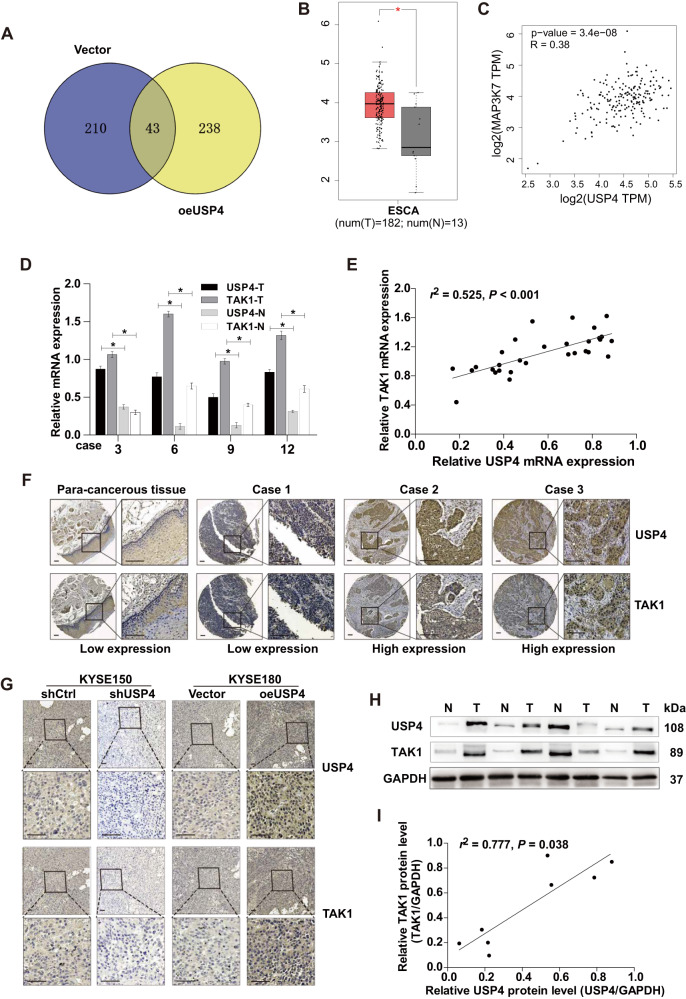
Close correlation between USP4 and TAK1 expression in ESCC. **A** The proteins that interacted with USP4 were identified using a combination of Co-IP and high-resolution LC-MS/MS analysis. The Venn diagram shows the number of binding partners of USP4 in the two groups. **B** Assessment and quantification of *TAK1* mRNA expression in EC primary tumor and normal tissues from the Gene Expression Profiling Interactive Analysis (GEPIA) database. **C** USP4 expression was positively correlated with TAK1 expression according to GEPIA data. **D** qRT-PCR analysis of *TAK1* mRNA levels in tumor and paired para-cancerous tissue specimens obtained from ESCC patients. **E** Assessment of the correlation between USP4 and TAK1 mRNA expression in ESCC specimens using Spearman’s correlation coefficient analysis. **F** Representative images of USP4 and TAK1 expression in ESCC tissues and para-cancerous tissues. **G** Assessment of the correlation between USP4 and TAK1 expression in specimens from ESCC xenograft model mice. **H, I** Western blotting analysis of the correlation between USP4 and TAK1 protein expression. *P < 0.05.

To unveil the molecular mechanism by which USP4 promotes ESCC progression, IP combined with MS analysis was performed in KYSE180 cells after USP4 overexpression, revealing multiple target genes with significant associations with USP4 (*P* < 0.05; fold change >1.5). IP-MS analysis and statistical filtering identified TAK1 as a candidate interactor of USP4 (Fig. 4A and Supplementary Table 4). Subsequently, we analyzed the differential expression of TAK1 between EC and noncancerous tissue samples based on the TCGA database. TAK1 expression was frequently upregulated in EC tissues compared to noncancerous tissues (Fig. 4B). Notably, TAK1 expression was positively correlated with the USP4 expression level (*R* = 0.38, Fig. 4C). Moreover, in 30 pairs of ESCC tissues and matched para-cancerous tissues, the mRNA levels of TAK1 were significantly increased in tissues with high USP4 expression and reduced in tissues with low USP4 expression (Fig. 4D). Correlation coefficient analysis demonstrated a positive correlation between USP4 and TAK1 expression (*r*^*2*^ = 0.525, *P* < 0.001, Fig. 4E). Subsequently, the relationship between USP4 and TAK1 protein expression was also detected in ESCC specimens compared to para-cancerous tissues by IHC analysis (Fig. 4F). Next, the effects of USP4 level alterations on the expression of TAK1 in ESCC mouse tissues were verified by USP4 knockdown or overexpression (Fig. 4G). Consistently, the protein levels of USP4 and TAK1 were increased in these 30 pairs of ESCC specimens, and a correlation was found between these proteins (*r*^*2*^ = 0.777, *P* = 0.038) (Fig. 4H, I). Together, these results proved a positive regulatory correlation between USP4 and TAK1.

### TAK1 as a direct target gene of USP4

**Fig. 5:**
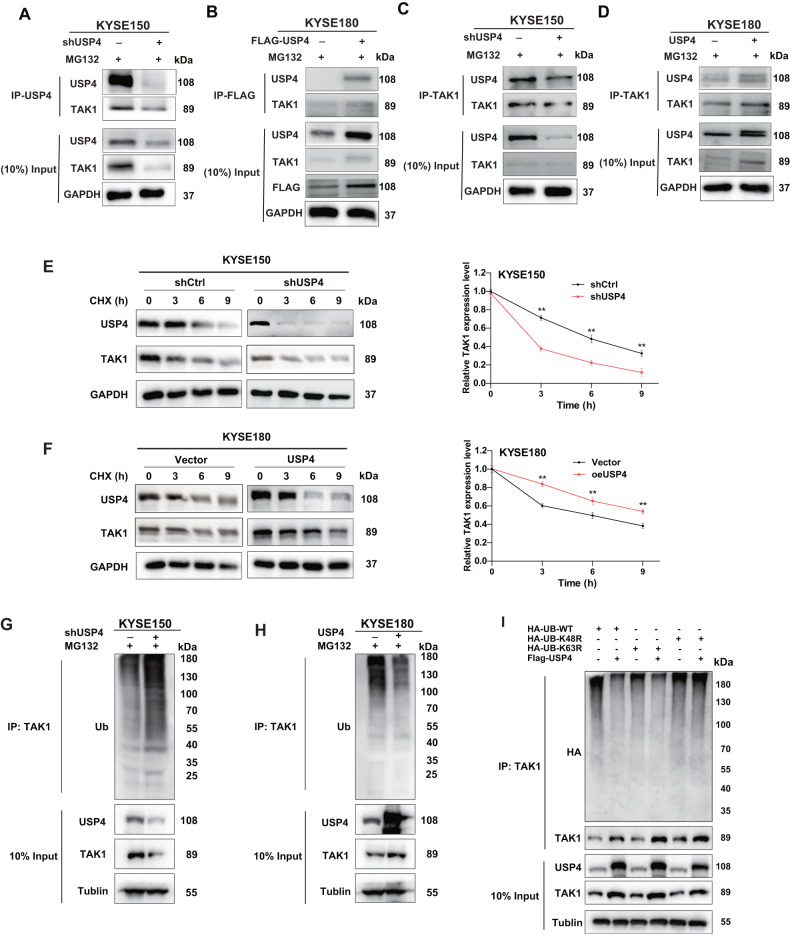
USP4 interacts with TAK1 in ESCC. **A**–**D** A Co-IP assay was used to verify the protein interaction between USP4 and TAK1. **E**, **F** At 0, 3, 6, and 9 h after CHX treatment of KYSE150 and KYSE180 cell lines, Western blotting was used to detect the levels of USP4 and TAK1 proteins to evaluate the effect of USP4 on the stability of TAK1 protein. **G**, **H** Ubiquitination of TAK1 was detected by ubiquitination assay and Western blotting. **I** HA-WT, K48R or K63R Ub were cotransfected with Flag-USP4 into HEK293T cells. After treatment with 20 μM MG132 for 6 h, cell lysates were subjected to a ubiquitination assay, and the ubiquitination level of TAK1 was detected by an anti-HA antibody. *P < 0.05, **P < 0.01, ***P < 0.001.

To verify the mass spectrometry results, a Co-IP assay was performed to test the reciprocal interaction between USP4 and TAK1 in ESCC cells. The results showed that USP4 interacted with TAK1 in both KYSE150 and KYSE180 cells (Fig. 5A–D). After treatment with CHX, the half-life of TAK1 was decreased by USP4 knockdown in KYSE150 cells, while USP4 overexpression in KYSE180 cells extended the half-life of TAK1 (Fig. 5E, F). To determine whether USP4 stabilizes TAK1 protein through its deubiquitination activity, we assessed the effect of USP4 on TAK1 ubiquitination in ESCC cells subjected to USP4 knockdown, MG132 treatment, or USP4 upregulation. As depicted in Fig. 5G, H, USP4 knockdown markedly increased the ubiquitination level of TAK1, and USP4 overexpression reduced the ubiquitination level of TAK1. We also performed a ubiquitination assay with K48R or K63R mutant (K48 or K63 lysine mutated to arginine). The results showed that USP4 could mainly remove the K48-linked ubiquitin chain from the TAK1 protein (Fig. 5I). Collectively, these results indicated that USP4 stabilizes TAK1 through a ubiquitination-dependent mechanism.

### TAK1 is required for USP4-related promotion of cell progression in ESCC

**Fig. 6:**
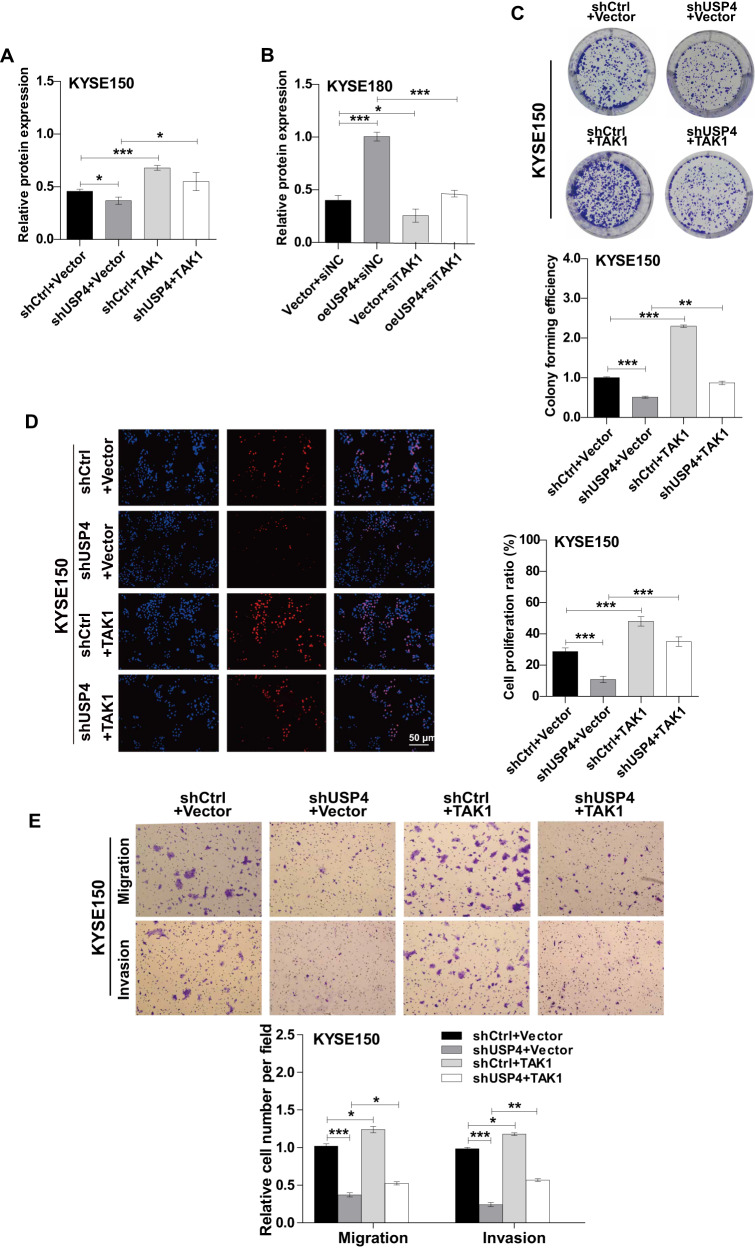
USP4 promoted the proliferation, migration, and invasion of ESCC cells by targeting TAK1. **A**, **B** Expression of USP4 and TAK1 proteins was detected by Western blotting. **C** Representative images and quantification of colony number by colony formation assay in KYSE150 cells. **D** Representative images (left panel) and quantification (right panel) of cell proliferation by EdU incorporation in KYSE150 cells. **E** Representative images (upper panel) and quantification (lower panel) of the capacity of migration and invasion by Transwell assays in KYSE150 cells. *P < 0.05, **P < 0.01, ***P < 0.001.

Based on the functional roles of USP4 in ESCC development and TAK1 expression, we speculated that USP4 promotes ESCC progression partially via TAK1. First, we ectopically expressed TAK1 in USP4-knockdown KYSE150 cells or knocked down TAK1 in USP4-overexpressing KYSE180 cells (Fig. 6A, B). The results of the cell function assays showed that overexpression of TAK1 could mostly restore the proliferation, migration, and invasion abilities of USP4-knockdown KYSE150 cells (Fig. 6C–E). Conversely, the function of USP4 in promoting the proliferation, migration, and invasion abilities of KYSE180 cells was largely reversed by the knockdown of TAK1 (Supplementary Fig. 2A-C). Taken together, these results indicate that USP4 accelerates ESCC cell progression by targeting TAK1.

### USP4 promotes ESCC progression via the MEK/ERK signaling pathway

**Fig. 7:**
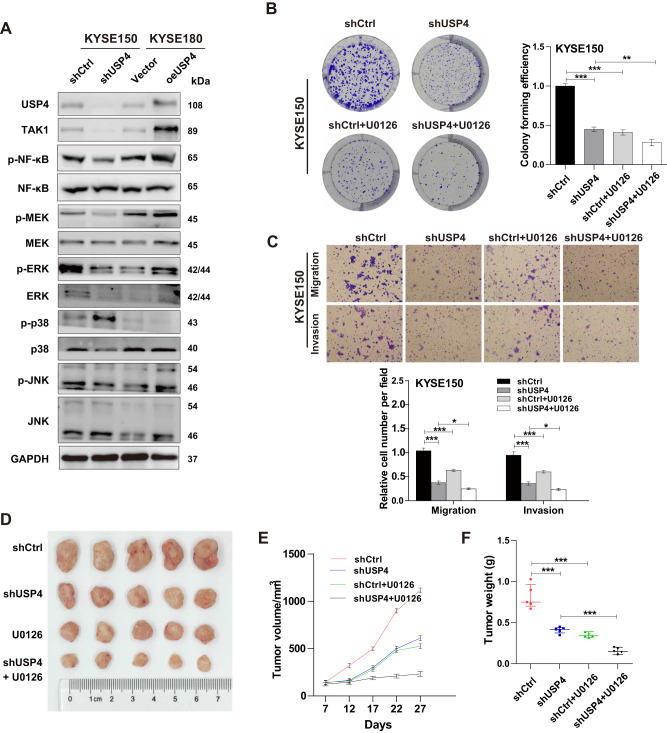
USP4 promotes ESCC progression by the MEK/ERK signaling pathway. **A** Expression of signaling pathway-related proteins was detected by Western blotting. **B** Representative images of the proliferation capacity of KYSE150 cells treated with U0126 by colony formation assay. **C** Representative images of the migration and invasion capacity of KYSE150 ESCC cells treated with U0126 by Transwell assays. **D** Combined treatment of U0126 can enhance the inhibitory effect of tumor growth caused by downregulation of USP4. **E** The volume of tumors was measured during the indicated periods. **F** The weights of tumors were determined and are represented as the mean ± SD. *P < 0.05, **P < 0.01, ***P < 0.001.

Finally, we investigated the mechanism by which USP4 promotes growth and metastasis in ESCC cells. Compared to the control groups, the USP4 knockdown group exhibited reduced phosphorylation levels of MEK, ERK, and NF-κB, while the USP4 overexpression group exhibited increased phosphorylation levels of MEK, ERK and NF-κB. However, we did not observe significant changes in MEK, ERK, NF-κB, p38, phosphorylated p38 (p-p38), JNK, and phosphorylated JNK (p-JNK) protein levels (Fig. 7A). These results showed that USP4 was involved in the regulation of the MEK/ERK signaling pathway. Furthermore, U0126, a specific inhibitor of MEK/ERK, was used to verify the involvement of the MEK/ERK signaling pathway in ESCC cells. Colony formation and Transwell assays demonstrated that U0126 suppressed the proliferation and metastasis abilities of KYSE150 cells, and simultaneous treatment with USP4 knockdown and U0126 significantly suppressed the above processes (Fig. 7B, C). Moreover, U0126 can partially attenuate the promotion of proliferation and metastasis by overexpression of USP4 in KYSE180 cells (Supplementary Fig. 3A, B). In vivo, treatment with U0126 further enhanced the suppression of tumor growth induced by downregulation of USP4 (Fig. 7D–F).

### Neutral red inhibits the proliferation and migration of ESCC cells

**Fig. 8:**
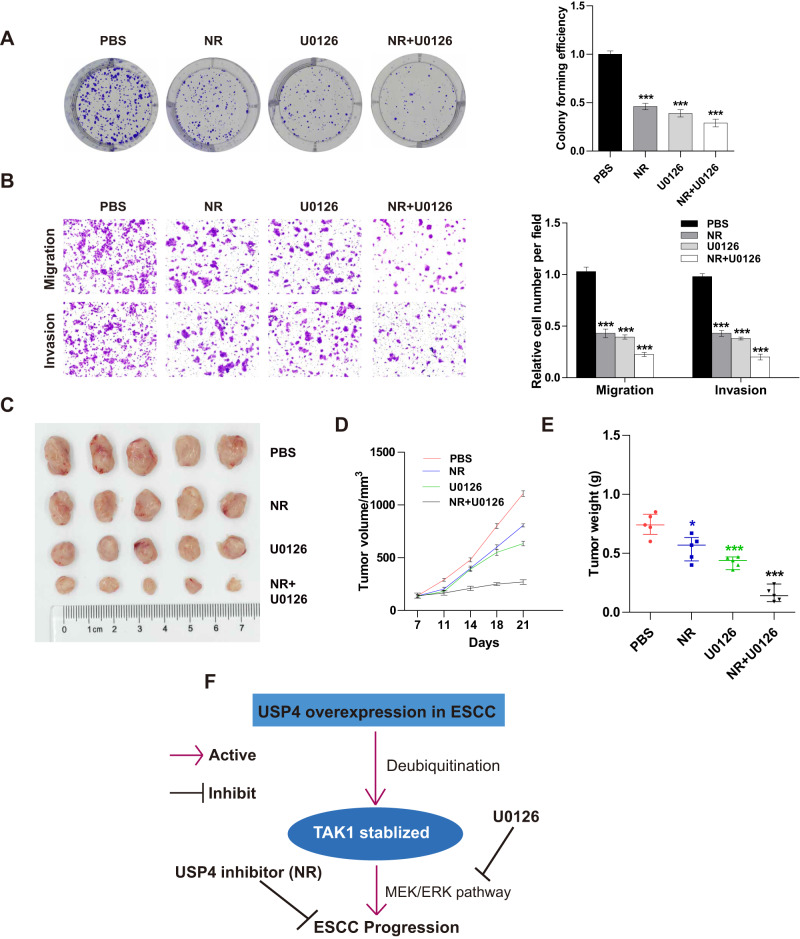
Neutral red inhibits cell progression in vitro and tumor cell growth in vivo. **A** KYSE150 cells were treated with NR, U0126, or a combination, and cell proliferation was detected by colony formation assay. **B** KYSE150 cells were treated with NR, U0126, or a combination, and cell migration and invasion were examined by Transwell assays. **C** Representative images of tumors excised from mice injected with PBS, NR, U0126, or a combination of NR and U0126. **D**, **E** The volume and weight of each tumor were measured during the indicated periods. **F** Schematic representation of the potential mechanism of USP4 in ESCC progression. *P < 0.05, ***P < 0.001.

Neutral red (NR) has been reported as a USP4 inhibitor in colon cancer [18]. We also expected that it would inhibit the proliferation and metastasis of ESCC cells. Colony formation ability was significantly inhibited when KYSE150 cells were treated with NR. Furthermore, the combination treatment of NR and U0126 clearly decreased cell proliferation (Fig. 8A). NR treatment also decreased the number of migrating and invading cells, and simultaneous treatment with NR and U0126 further suppressed the migration and invasion of cancer cells (Fig. 8B). The xenograft tumor models in mice were generated using KYSE150 cells. PBS (control), NR (50 mg/kg), or U0126 (15 mg/kg) was injected intraperitoneally every 3 days. Compared to the control, NR or U0126 treatment can significantly inhibit the growth of the tumors, and the combination of NR and U0126 can exert greater inhibitory effects (Fig. 8C–E).

Collectively, these results indicated that USP4 stabilizes the TAK1 protein through deubiquitination, thereby accelerating cell growth and metastasis via regulation of the MEK/ERK signaling pathway in ESCC cells (Fig. 8F).

## Discussion

The present study demonstrated enhanced USP4 expression in ESCC tissue specimens and cell lines and revealed that USP4 overexpression promoted ESCC progression via regulation of cell proliferation, migration, and invasion in vitro and in vivo. We identified TAK1 as a candidate target protein for USP4. Further mechanistic studies showed that USP4 regulates the ESCC cellular phenotype by regulating the ubiquitination level of the TAK1 protein via the MEK/ERK signaling pathway. Treatment with the inhibitors of USP4 (neutral red) and MEK/ERK (U0126) can inhibit the progression of ESCC cells in vivo, and can also inhibit tumorigenesis in vitro. These results suggest that USP4 plays an important role in ESCC malignancy, and it may serve as a novel prognostic marker for ESCC.

USP4 is a DUB with USP cysteine (Cys) and histidine (His) boxes and deubiquitinating activity [19]. Reportedly, USP4 can be used as a prognostic biomarker because it is upregulated in diverse malignancies, including lung cancer, multiple myeloma, and pancreatic cancer [19–21]. Li et al. revealed that USP4 promoted lung cancer cell stemness by stabilizing Twist1 protein expression [22]. In the present study, upregulated USP4 expression was observed in ESCC tissue and ESCC cell lines, and its expression in ESCC patients was positively correlated with large tumor size, high invasion depth, lymph node metastasis, and poor patient survival, suggesting an essential role of USP4 in ESCC progression. Mounting evidence from in vitro and in vivo studies has indicated that USP4 has a crucial role in multiple cellular and biological processes, and USP4 overexpression enhances the proliferation and migration abilities of cancer cells [21, 23, 24]. This phenomenon was also confirmed in the current study. In contrast, depletion of USP4 significantly inhibited proliferation, migration, and invasion abilities in vitro and suppressed tumor growth and metastasis in vivo. Thus, USP4 is a promising tumor biomarker and therapeutic target for ESCC. Interestingly, USP4 was reported to exhibit a tumor suppressor role in certain cancers, including breast cancer and head and neck squamous cell carcinoma [11, 25]. Moreover, downregulated USP4 inhibited the expression of SMAD4 by its deubiquitination and promoted EC progression [26]. USP4 may exert completely opposite effects in the same cancer, possibly because the roles of USP4 in cancer are controlled by a complex network of pathways that vary in different cell types.

The exact mechanism by which USP4 induces these alterations requires further study. In a previous study on breast cancer, USP4 was shown to promote TGF-β signaling and to cooperate with AKT signaling to promote cancer cell EMT, migration, and invasion [27]. Conversely, USP4 may negatively regulate the NF-κB signaling pathway by targeting multiple signaling molecules, including TRAF2 and TRAF6 [28]. TAK1 represents a serine/threonine kinase belonging to the MAPK family, which is essential in TNFα-induced activation of the NF-κB, JNK, and p38 pathways [29]. Accumulating studies have highlighted the involvement of TAK1 in cancer progression [30, 31]. Reportedly, the inhibition of TAK1 kinase activity dramatically promoted cell apoptosis, thereby markedly enhancing tumor sensitivity to chemotherapeutics and irradiation [32]. However, the role of TAK1 in ESCC progression remains unclear.

As demonstrated above, TAK1 expression was upregulated in ESCC tissues. Functional experiments further indicated that depletion of TAK1 reduced the proliferation, migration, and invasion abilities of ESCC cells, while overexpression exerted a promoting effect. In agreement with a previous study showing that USP4 acts as a deubiquitinase for TAK1 [33], the present study also indicated that USP4 knockdown suppressed TAK1 deubiquitination, but overexpression enhanced its deubiquitination. Importantly, USP4 dramatically accelerates the malignant phenotype of ESCC cells by targeting TAK1 in ESCC, suggesting that the USP4–TAK1 axis plays an important role in ESCC malignant progression. The MAPK signaling pathway is a major intracellular signaling pathway that facilitates ESCC progression and was chosen for testing in the present study [31]. Our results showed that USP4 positively regulates the MER/ERK signaling pathway via a TAK1-dependent process. U0126, a specific inhibitor of MEK/ERK, counteracted the promoting effects of USP4 overexpression on cell proliferation, metastasis, and tumor growth. Overall, these results suggest that the USP4–TAK1 axis plays pro-oncogenic roles in ESCC progression through regulation of the MEK/ERK signaling pathway. However, a more detailed molecular mechanism of this phenomenon and the clinical transformation of this pathway in ESCC need to be explored further in the future.

In summary, USP4 expression is upregulated in ESCC tissues and cell lines and in turn regulates the TAK1 protein via the ubiquitin‒proteasome system to promote the progression of ESCC. Therefore, USP4 may be a potential molecular therapeutic target for ESCC treatment.

### Supplementary information

Supplementary Figure legend

Supplementary Figure 1

Supplementary Figure 2

Supplementary Figure 3

Supplementary Tables

Checklist

Original Data File

## Data Availability

All data generated or analyzed during this study are included in this published article and its Supplementary Information files.
