# Supplementary material for: USP4 promotes the proliferation, migration, and invasion of esophageal squamous cell carcinoma by targeting TAK1
Source: Cell Death Dis. 2023 Nov 10;14(11):730. doi: 10.1038/s41419-023-06259-0 (PMC10638297; doi:10.1038/s41419-023-06259-0)
Supplement: Supplementary file 1 — Supplementary Figure legend [file 41419_2023_6259_MOESM1_ESM.docx]

**Supplementary Figure Legends**

**Supplementary Figure 1. The expression of USP4 in KYSE150 and KYSE180 cells after transfection with lentiviral solution**. **A, B** The mRNA and protein levels of USP4 in KYSE150 cells after transfection with shUSP4. **C, D** The mRNA and protein levels of USP4 in KYSE180 cells after transfection with oeUSP4. **E** The expression of USP4 in different xenograft tumor tissues was detected by IHC staining. Each experiment was performed in duplicate and repeated 3 times. ****P* < 0.001.

**Supplementary Figure 2. USP4 promoted the proliferation, migration, and invasion in KYSE180 cells. A** Representative images and quantification of colony number by colony formation assay. **B** Representative images and quantification of cell proliferation by EdU incorporation. **C** Representative images and quantification of the capacity of migration and invasion by Transwell assay. **P* < 0.05, ***P* < 0.01, ****P* < 0.001.

**Supplementary Figure 3. The effect of U0126 on the progression of KYSE180 cells** **transfected with oeUSP4. A** Representative images of the effect of U0126 on the proliferation capacity of KYSE180 cells transfected with oeUSP4 by colony formation assays. **B** Representative images of the effect of U0126 on the migration and invasion capacity of KYSE180 cells transfected with oeUSP4 by Transwell assays. **P* < 0.05, ***P* < 0.01, ****P* < 0.001.
