# Supplementary figures and images for: USP4 promotes the proliferation, migration, and invasion of esophageal squamous cell carcinoma by targeting TAK1
Source: Cell Death Dis. 2023 Nov 10;14(11):730. doi: 10.1038/s41419-023-06259-0 (PMC10638297; doi:10.1038/s41419-023-06259-0)

Supplementary Figure 1

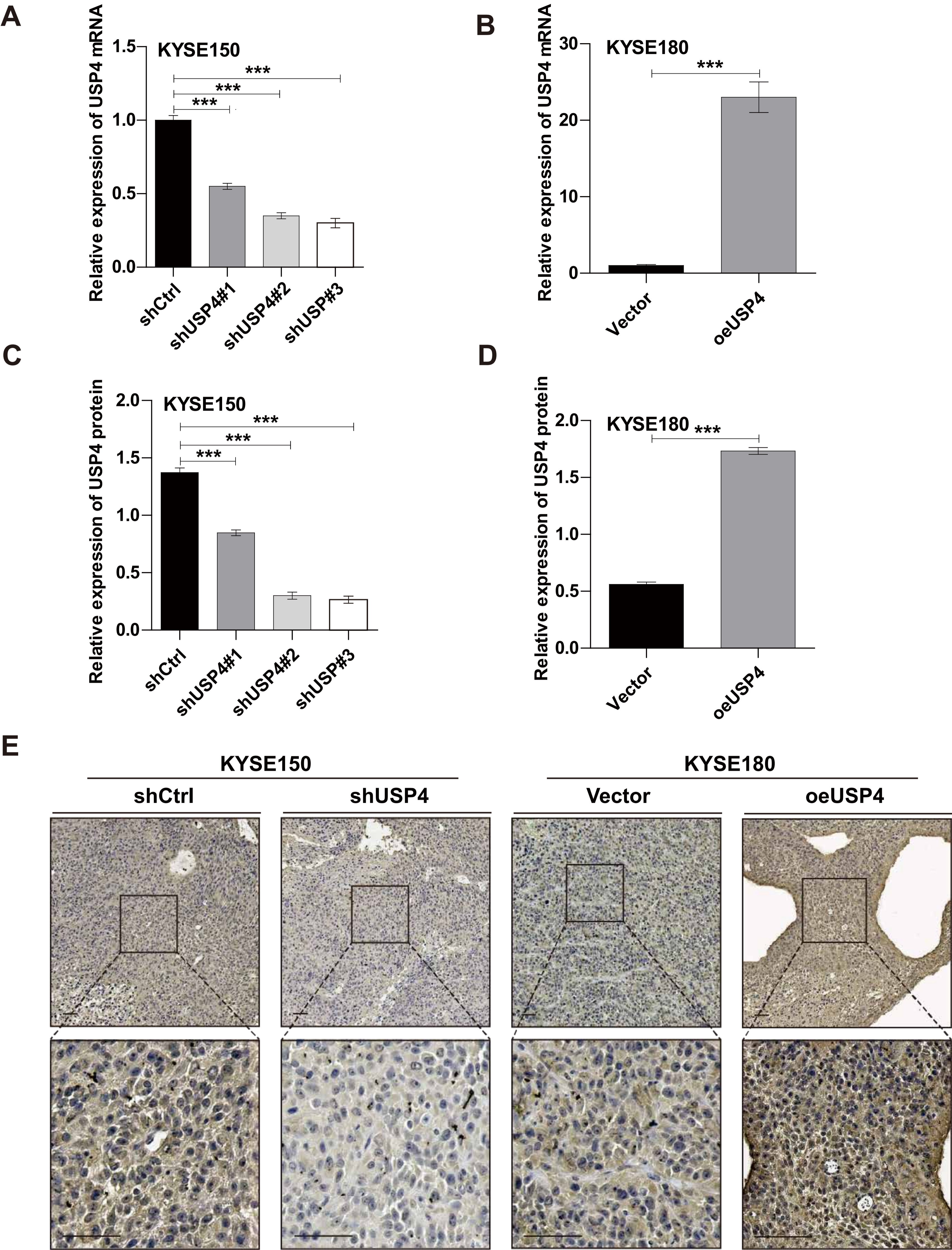

Supplement: Supplementary file 2 — Supplementary Figure 1 [file 41419_2023_6259_MOESM2_ESM.pdf]

Supplementary Figure 2

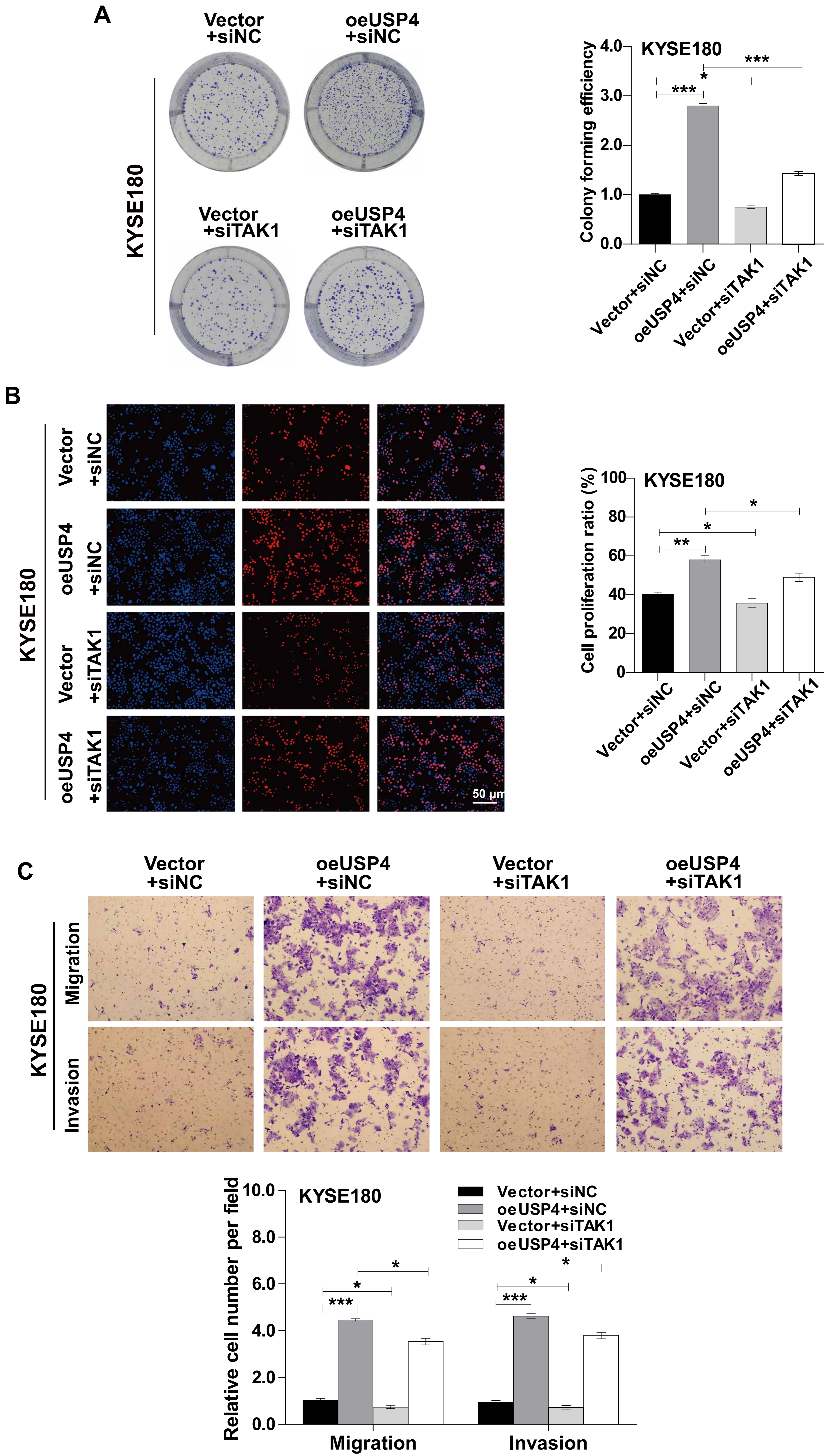

Supplement: Supplementary file 3 — Supplementary Figure 2 [file 41419_2023_6259_MOESM3_ESM.pdf]

Supplementary Figure 3

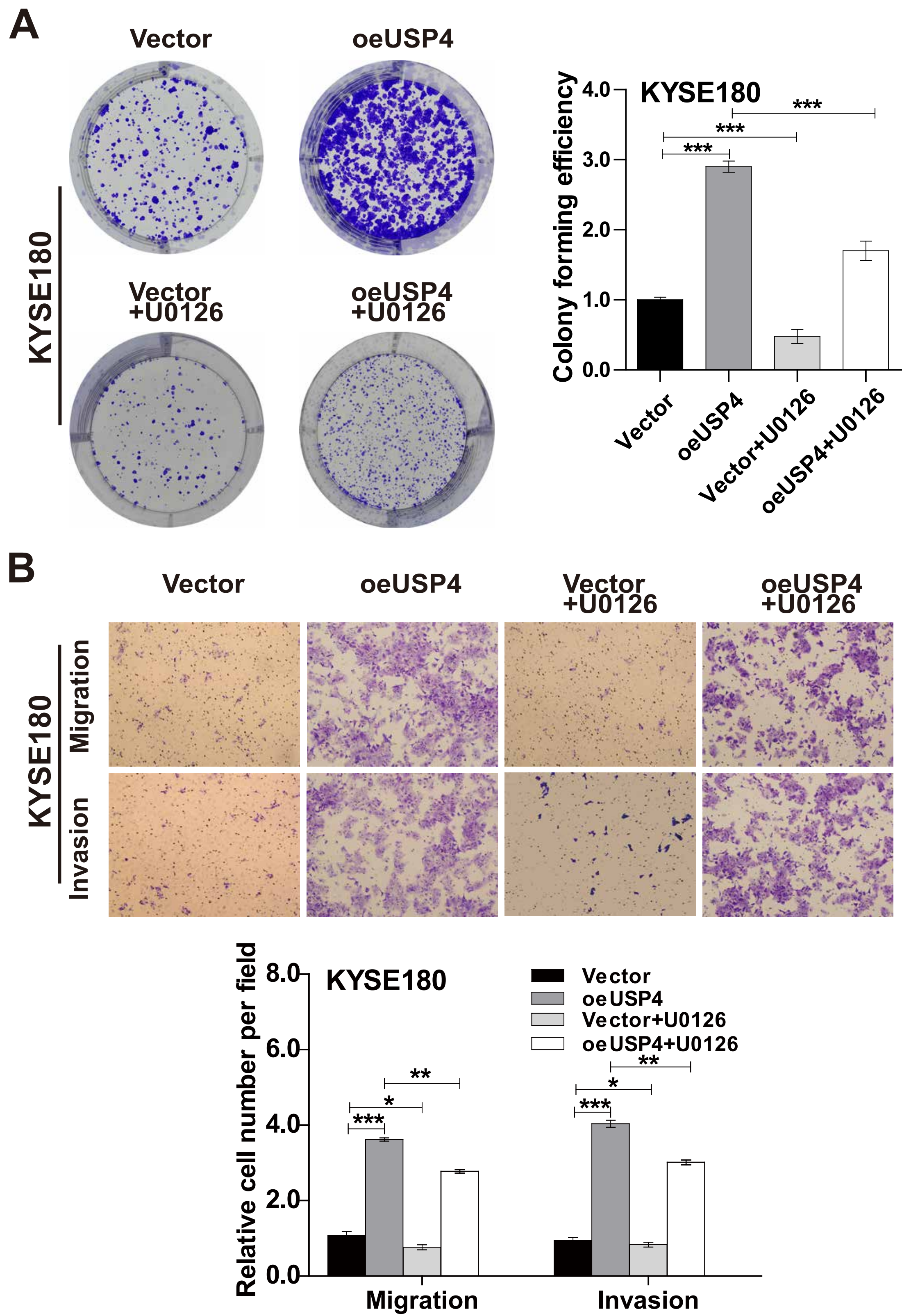

Supplement: Supplementary file 4 — Supplementary Figure 3 [file 41419_2023_6259_MOESM4_ESM.pdf]
