# Supplementary material for: USP4 promotes the proliferation, migration, and invasion of esophageal squamous cell carcinoma by targeting TAK1
Source: Cell Death Dis. 2023 Nov 10;14(11):730. doi: 10.1038/s41419-023-06259-0 (PMC10638297; doi:10.1038/s41419-023-06259-0)
Supplement: Supplementary file 5 — Supplementary Tables [file 41419_2023_6259_MOESM5_ESM.docx]

**Supplementary Table 1.** Sequences of USP4 shRNA and TAK1 siRNA

| **Target gene** |  | **Sequences** |
| --- | --- | --- |
| USP4 shNC |  | Sequence unknown (synthesized by GeneChem) |
| USP4 shRNA1 |  | AAAAGGGTGAACATAACCTATTTCTTGGATCCAAGAAATAGGTTATGTTCACCC |
| USP4 shRNA2 |  | AAAAGCGTGGAATAAACTACTAATTGGATCCAATTAGTAGTTTATTCCACGC |
| USP4 shRNA3 |  | AAAAGCAAAGTCGAGGTGTATTTGTTGGATCCAACAAATACACCTCGACTTTGC |
| TAK1 siRNA |  | GGUAGUAAUUACAGUGAAATT  UUUCACUGUAAUUACUACCTT |

**Abbreviation**: USP4, ubiquitin-specific protease 4; TAK1, transforming growth factor-β-activated kinase 1.

**Supplementary Table 2.** Primer sequences for qRT-PCR

| **Gene** | **Primer** | **Sequence** |
| --- | --- | --- |
| USP4 | Forward (5’ to 3’) | CGCCTAGCAGAATTTTTACTACCTC |
|  | Reverse (5’ to 3’) | TGAGAAAGTAGTCAGTCAGTGGTGC |
| TAK1 | Forward (5’ to 3’) | GGAGATCGAGGTGGAAGAGG |
|  | Reverse (5’ to 3’) | GGTTCACACGGGATAACTGC |
| GAPDH | Forward (5’ to 3’) | TCCGTGGAGAAGAGCTACGA |
|  | Reverse (5’ to 3’) | GTACTTGCGCTCAGAAGGCG |

**Supplementary Table 3.** Antibodies used in the current study.

| **Primary antibody** | **Product** | **Western Blot ^a^** | **IP ^b^** | **IHC ^c^** |
| --- | --- | --- | --- | --- |
| Rabbit anti-USP4 | #2651^d^ | 1:1000 |  |  |
| Rabbit anti-USP4 | ab245654 **^e^** |  | 2-10 µg/mg |  |
| Mouse anti-USP4 | 66822-1-Ig ^f^ |  |  | 1:100 |
| Rabbit anti-TAK1 | #5206 ^d^ | 1:1000 | 1:50 |  |
| Mouse anti-TAK1 | 67707-1-Ig ^f^ |  |  | 1:100 |
| anti-Flag-tag | F3165 ^g^ | 0.5-10µg/ml |  |  |
| anti-Ub | sc-8017 ^h^ | 1:500 |  |  |
| anti-HA | sc-57592 ^h^ | 1:500 |  |  |
| anti-p-MEK | #9154 ^d^ | 1:1000 |  |  |
| anti-MEK | #8727 ^d^ | 1:1000 |  |  |
| anti-p-ERK1/2 | 80031-1-RR ^f^ | 1:1000 |  |  |
| anti-ERK1/2 | 51068-1-AP ^f^ | 1:1000 |  |  |
| anti-p-NF-κB | #3039 ^d^ | 1:1000 |  |  |
| anti-NF-κB | #8242 ^d^ | 1:1000 |  |  |
| anti-p-p38 | 28796-1-AP ^f^ | 1:2000 |  |  |
| anti-p38 | GTX110720 ^i^ | 1:1000 |  |  |
| anti-p-JNK | 80024-1-RR ^f^ | 1:2000 |  |  |
| anti-JNK | 66210-1-Ig ^f^ | 1:10000 |  |  |
| anti-GAPDH | ab9485 ^e^ | 1:2500 |  |  |
| anti-Tublin | #2148 ^d^ | 1:1000 |  |  |

a. Antibody dilution for western blotting

b. Antibody dilution for immunoprecipitation

c. Antibody dilution for immunohistochemistry staining

d. Cell Signaling Technology

e. Abcam

f. Proteintech

g. Sigma

h. Santa Cruz Biotechnology

i. Genetex

**Supplementary Table 4**. The binding proteins of USP4 were identified by Co-IP/MS.

| **Protein name** | **molecular weight (KDa)** | **Sequence coverage (%)** | **Number of specific peptides** | **abundance** |
| --- | --- | --- | --- | --- |
| MYH9 | 226.4 | 44 | 66 | 9.72E+08 |
| ACTB | 41.7 | 77 | 1 | 1.71E+09 |
| ACTG1 | 41.8 | 77 | 1 | 2.40E+06 |
| USP4 | 108 | 20 | 14 | 5.03E+07 |
| MYH14 | 227.7 | 18 | 24 | 5.47E+07 |
| HNRNPH1 | 49.2 | 47 | 9 | 3.21E+08 |
| MYO1D | 116.1 | 28 | 25 | 1.17E+08 |
| TAK1 | 89 | 36 | 23 | 8.44E+07 |
| RTCB | 55.2 | 44 | 26 | 1.04E+08 |
| DDX1 | 82.4 | 37 | 24 | 1.22E+08 |
| THRAP3 | 108.6 | 26 | 23 | 1.87E+08 |
| HNRNPH2 | 49.2 | 35 | 4 | 2.26E+07 |
| VCP | 89.3 | 28 | 18 | 8.68E+07 |
| DDX3X | 73.2 | 30 | 14 | 7.97E+07 |
| MYH10 | 228.9 | 22 | 23 | 5.88E+07 |

**Abbreviation**: Co-IP/MS: Co-immunoprecipitation coupled to mass spectrometry
