# Supplementary material for: USP4 promotes the proliferation, migration, and invasion of esophageal squamous cell carcinoma by targeting TAK1
Source: Cell Death Dis. 2023 Nov 10;14(11):730. doi: 10.1038/s41419-023-06259-0 (PMC10638297; doi:10.1038/s41419-023-06259-0)
Supplement: Supplementary file 7 — Original Data File [file 41419_2023_6259_MOESM7_ESM.pdf]

Figure 1D

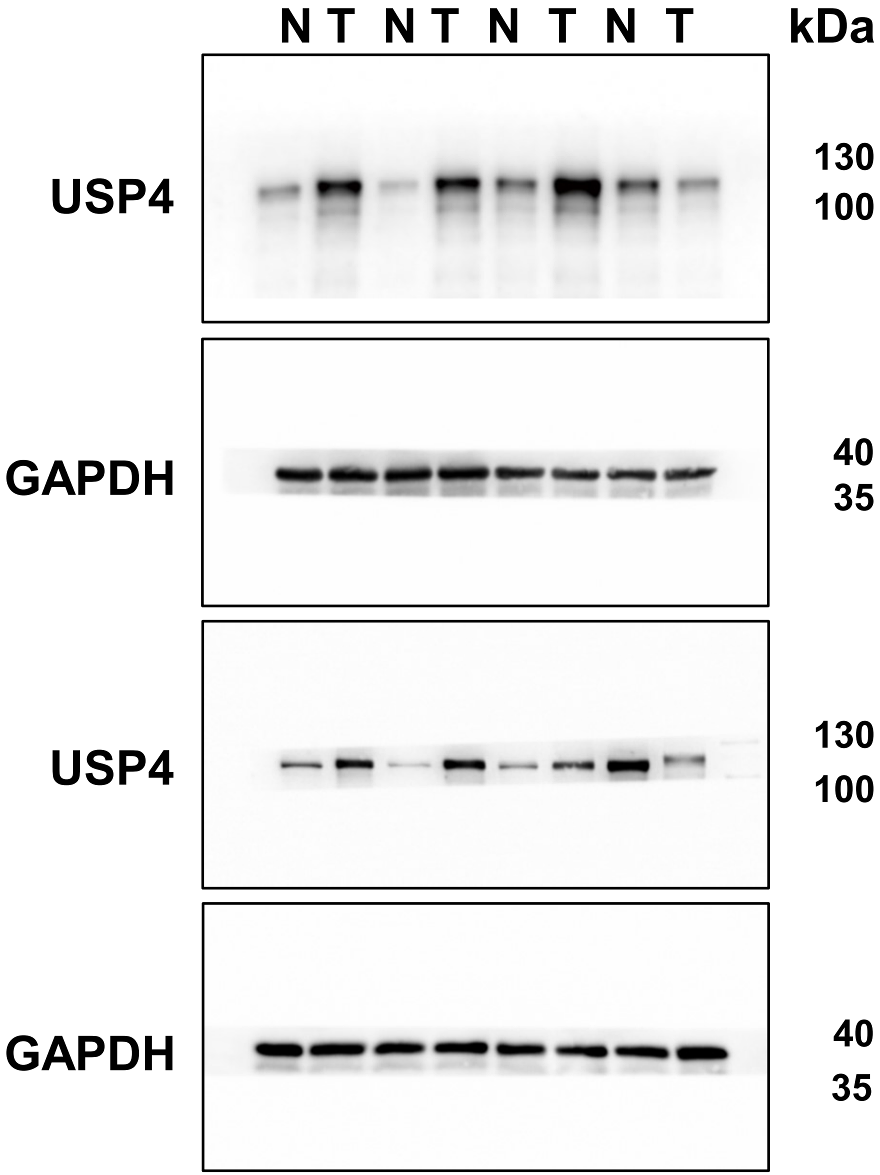

Figure 1H

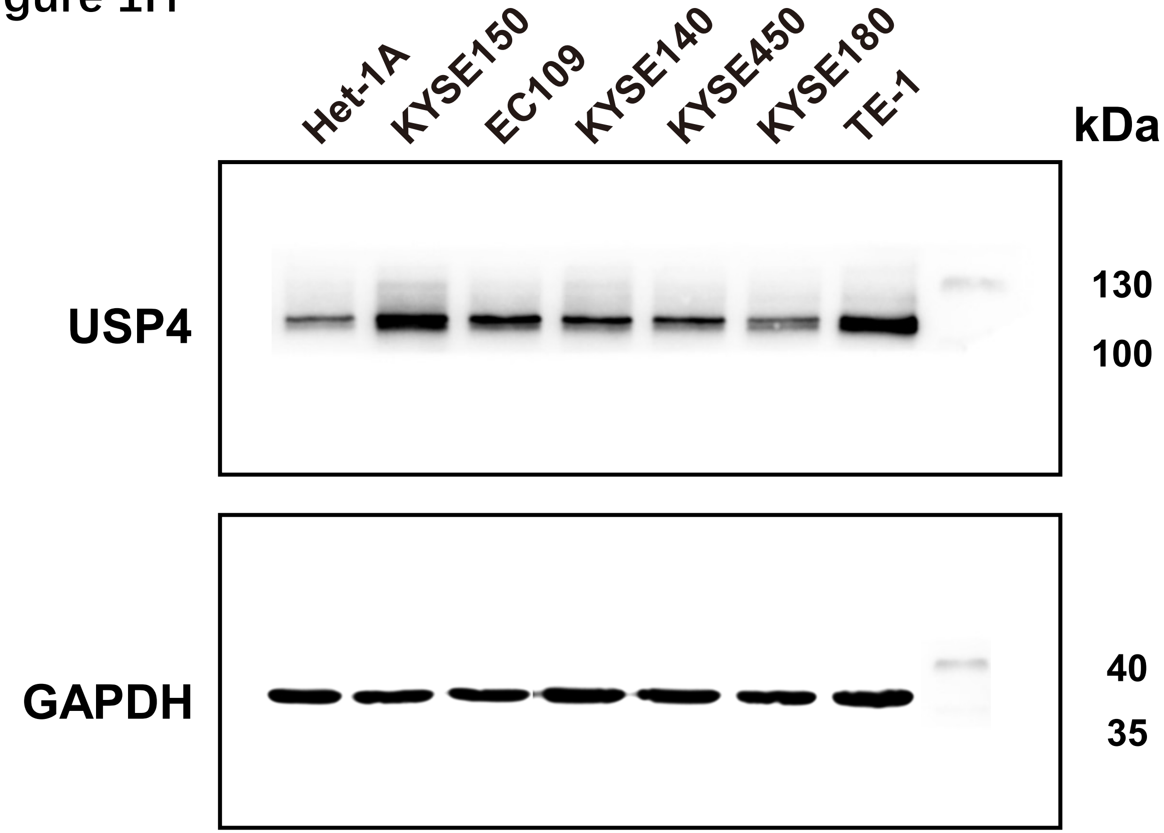

Figure 2A

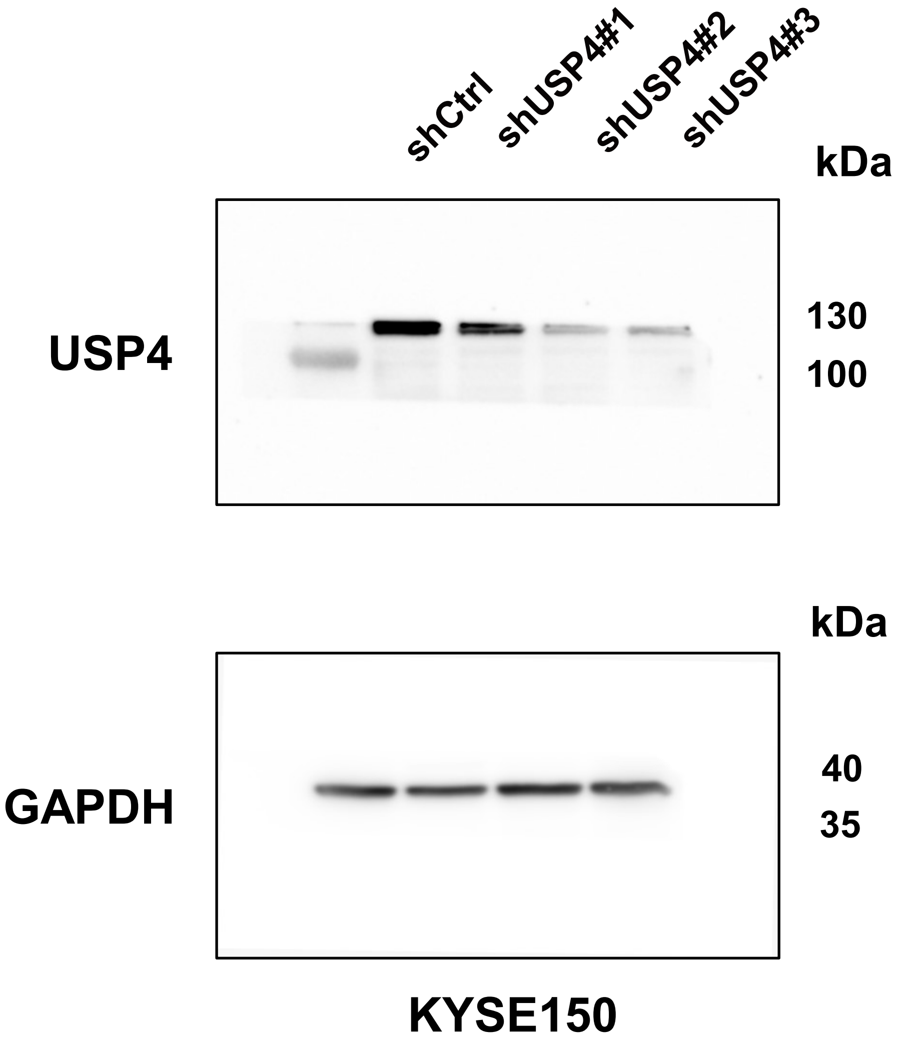

Figure 2B

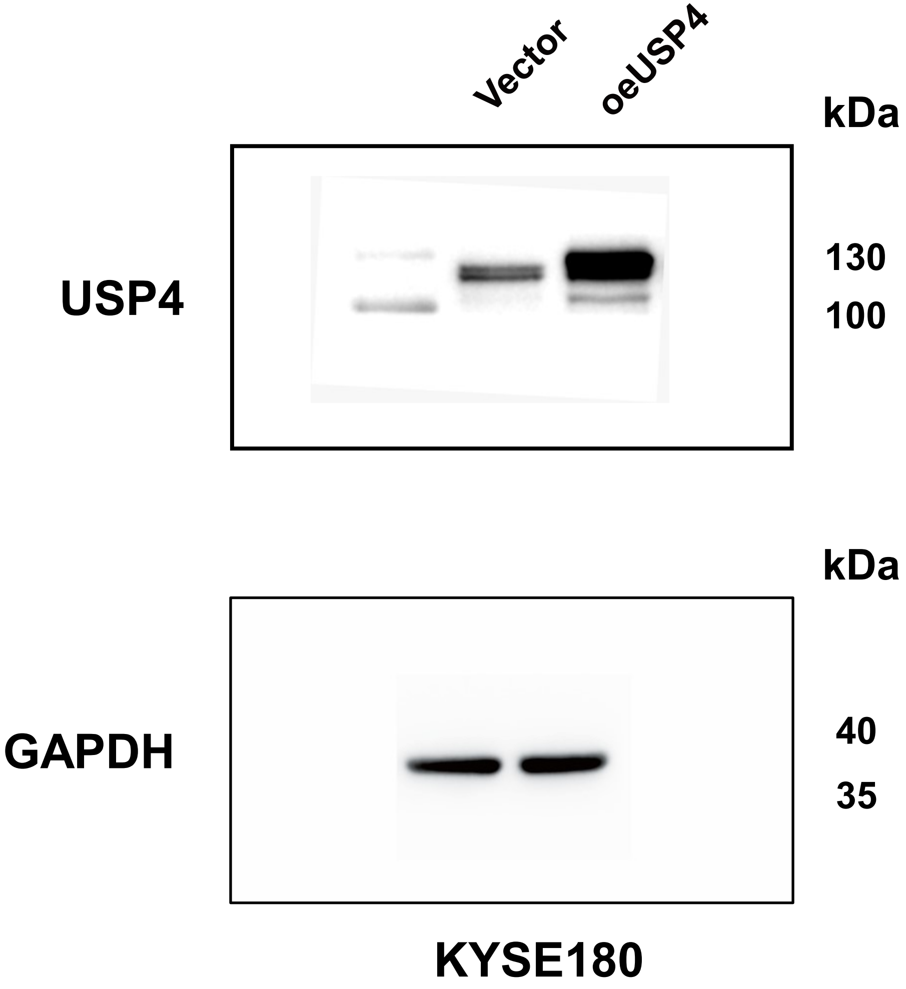

Figure 4H

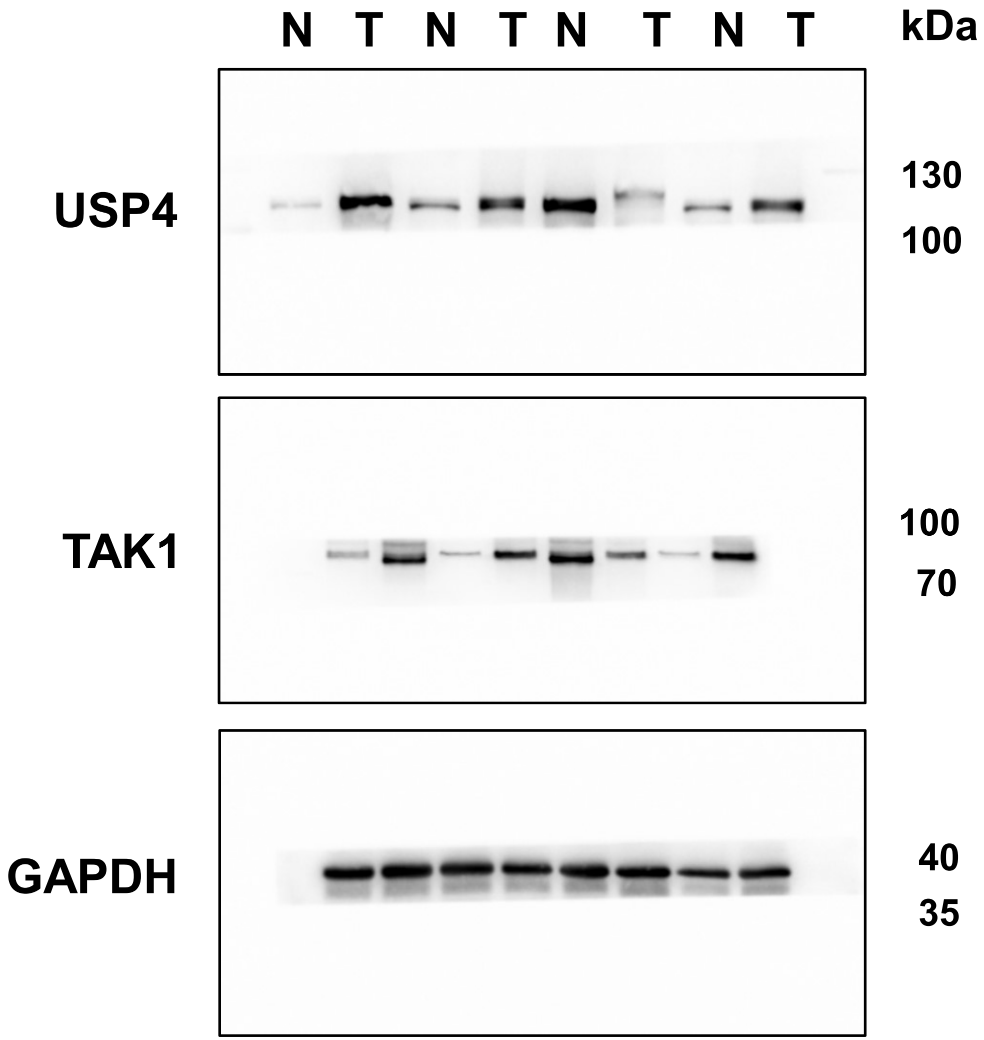

Figure 5A

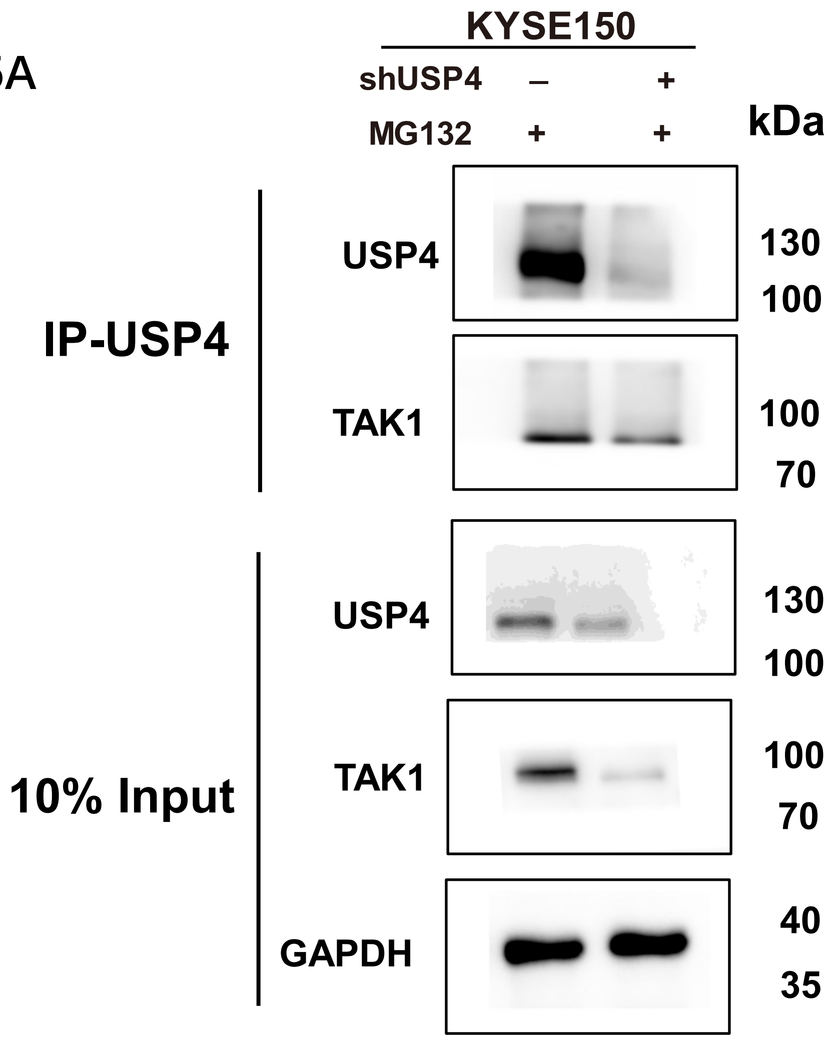

Figure 5B

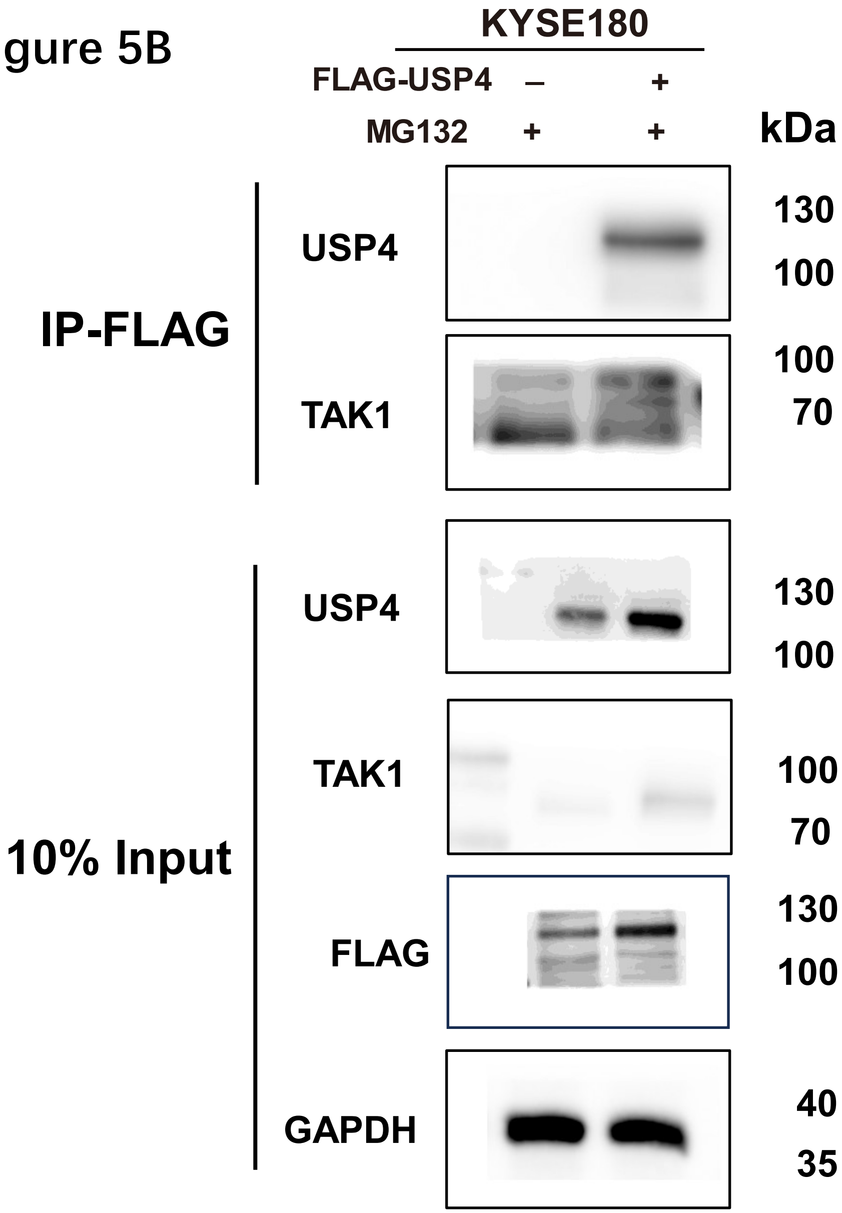

Figure 5C

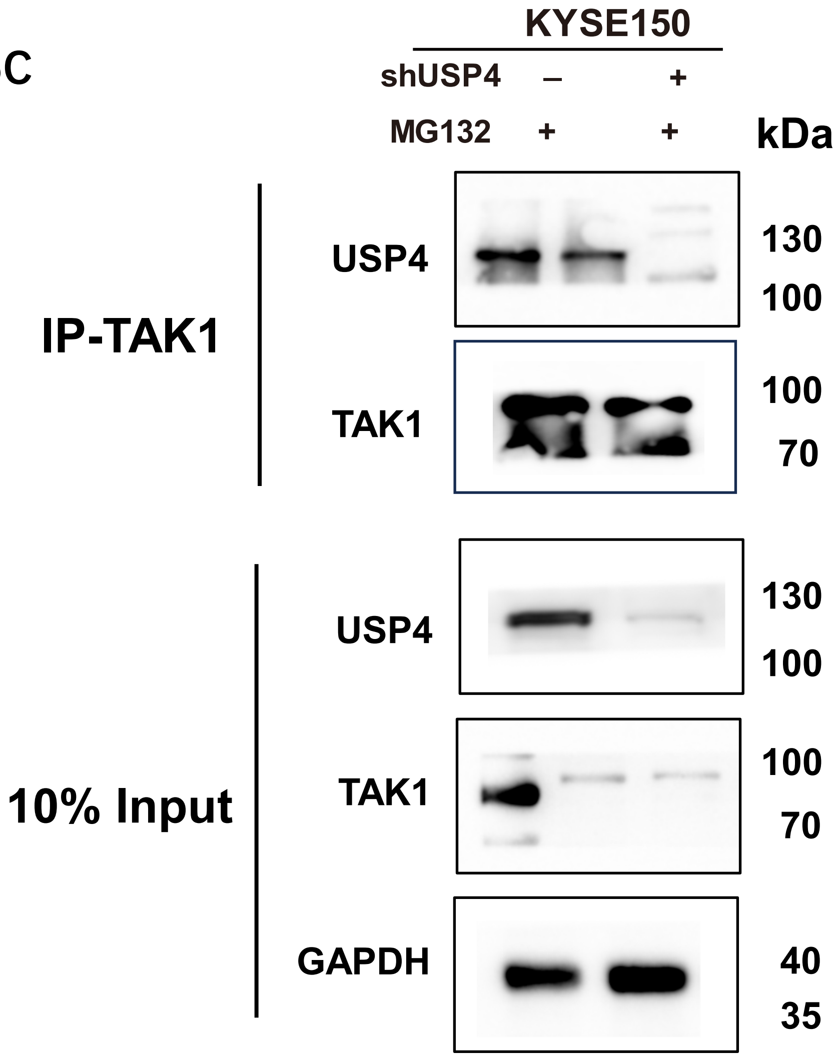

Figure 5D

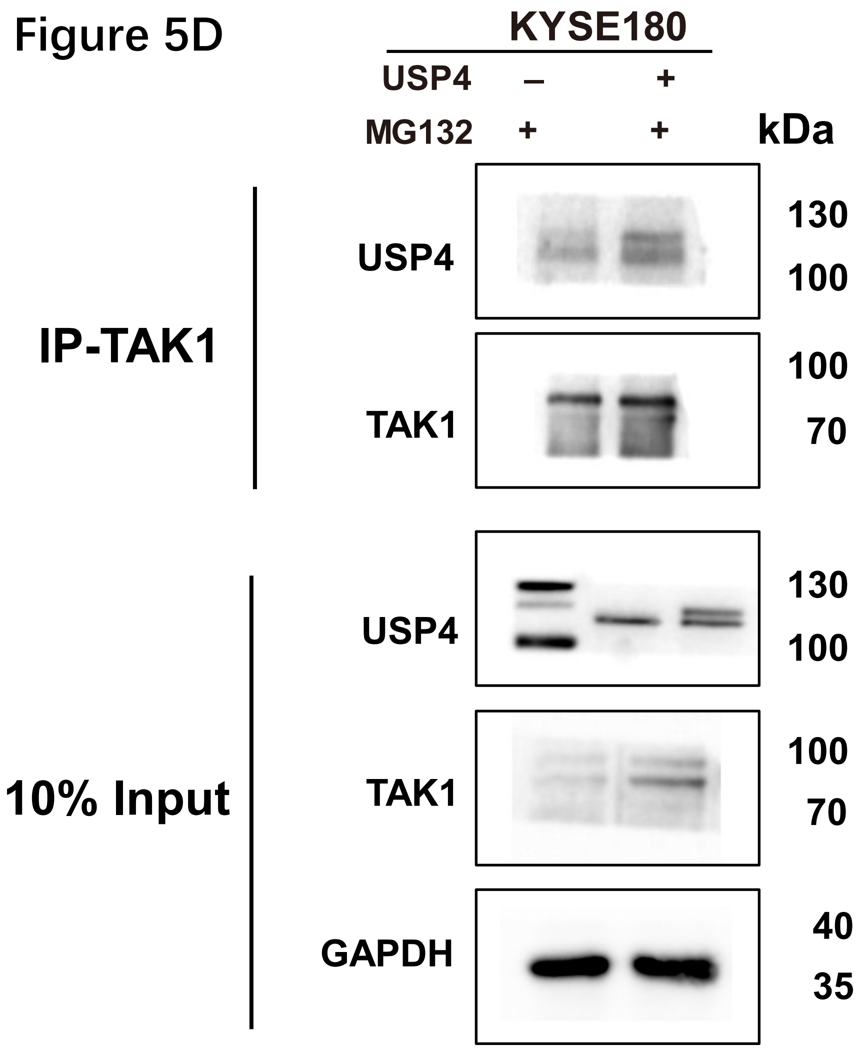

Figure 5E-F

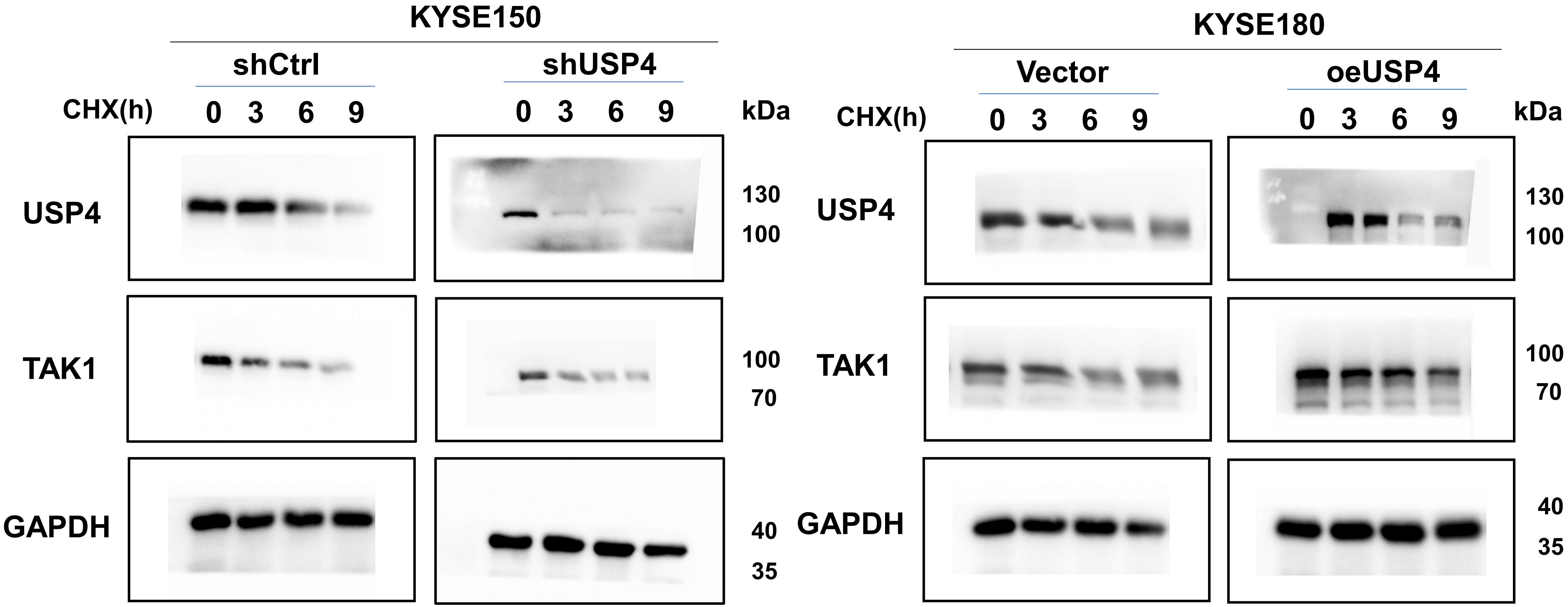

KYSE150
shCtrl
shUSP4
CHX(h)
0 3 6 9
0 3 6 9
kDa
USP4
TAK1
GAPDH
130
100
100
70
40
35
KYSE180
Vector
oeUSP4
CHX(h)
0 3 6 9
0 3 6 9
kDa
USP4
TAK1
GAPDH
130
100
100
70
40
35

Figure 5G-H

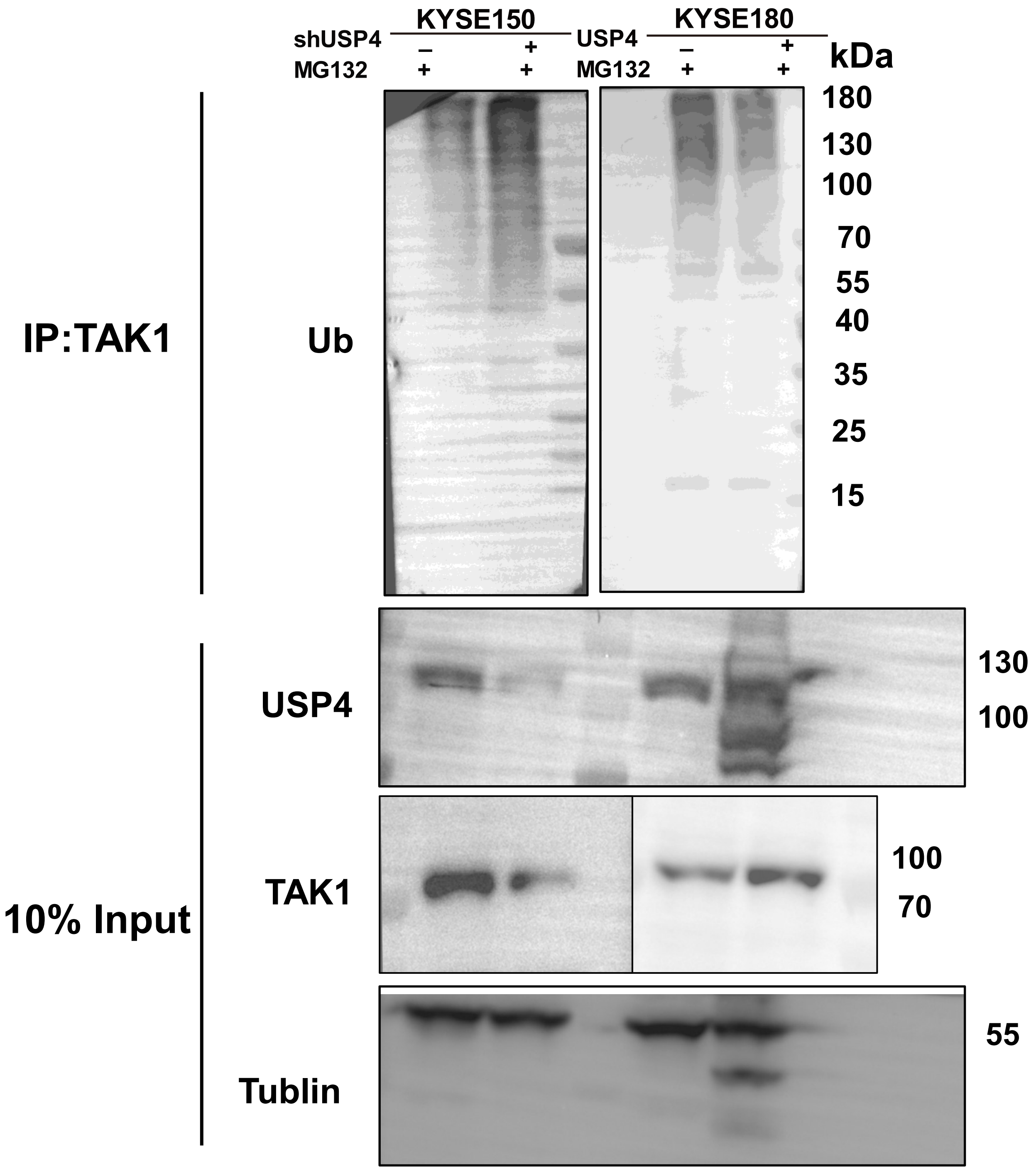
KYSE150
shUSP4
MG132
–
+
+
+
KYSE180
USP4
MG132
–
+
+
+
kDa
180
130
100
70
55
40
35
25
15
IP:TAK1
Ub
10% Input
USP4
130
100
TAK1
100
70
Tublin
55

Figure 5I

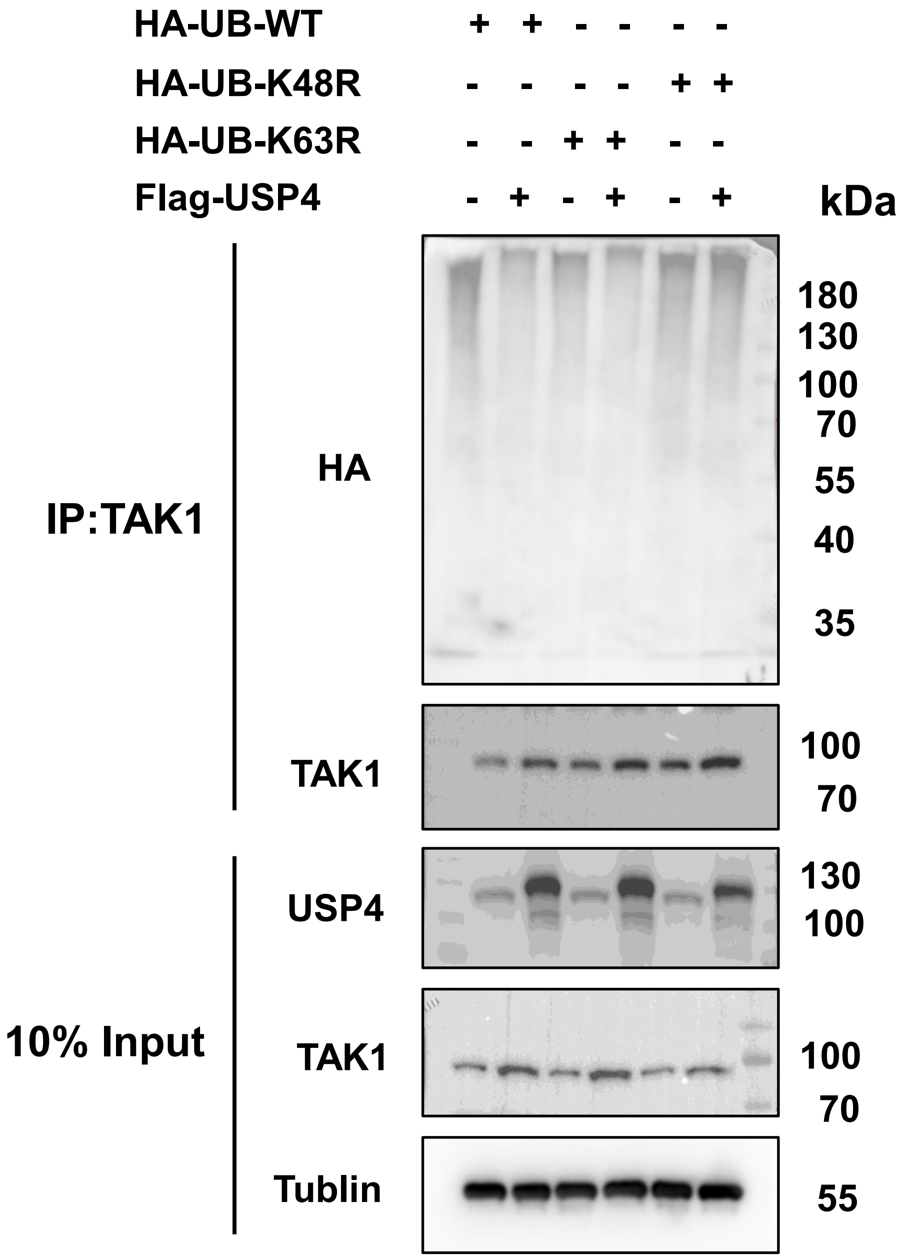

HA-UB-WT + + - - - -
HA-UB-K48R - - - - + +
HA-UB-K63R - - + + - -
Flag-USP4 - + - + - +
kDa
IP:TAK1
HA
180
130
100
70
55
40
35
TAK1
100
70
10% Input
USP4
130
100
TAK1
100
70
Tublin
55

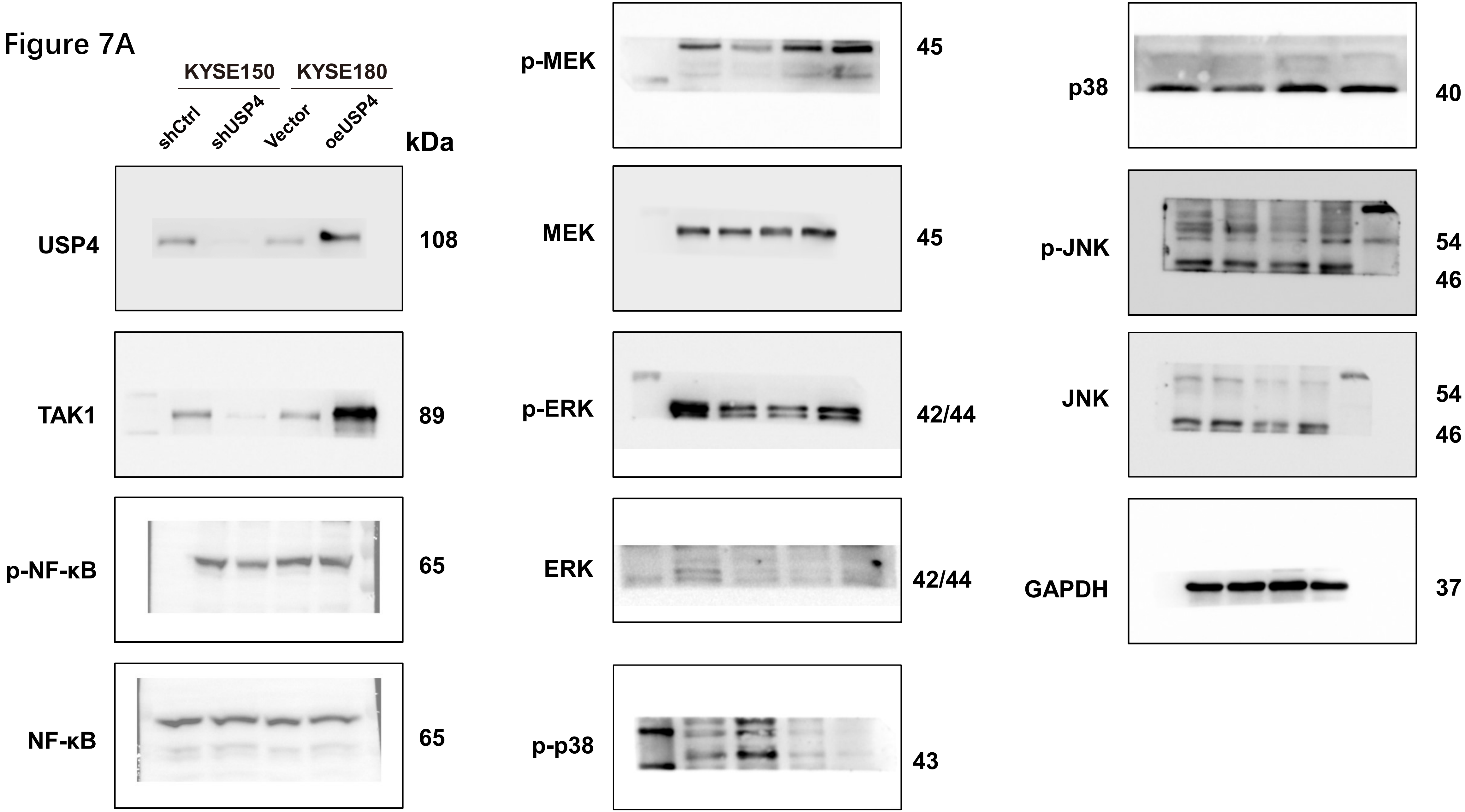

Figure 7A
KYSE150
KYSE180
shCtrl
shUSP4
Vector
oeUSP4
kDa
USP4
108
TAK1
89
p-NF-κB
65
NF-κB
65
p-MEK
45
MEK
45
p-ERK
42/44
ERK
42/44
p-p38
43
p38
40
p-JNK
54
46
JNK
54
46
GAPDH
37
